# Quantitative trait loci for resistance to *Flavobacterium psychrophilum* in rainbow trout: effect of the mode of infection and evidence of epistatic interactions

**DOI:** 10.1186/s12711-018-0431-9

**Published:** 2018-11-16

**Authors:** Clémence Fraslin, Nicolas Dechamp, Maria Bernard, Francine Krieg, Caroline Hervet, René Guyomard, Diane Esquerré, Johanna Barbieri, Claire Kuchly, Eric Duchaud, Pierre Boudinot, Tatiana Rochat, Jean-François Bernardet, Edwige Quillet

**Affiliations:** 1 GABI, INRA, AgroParisTech, Université Paris-Saclay, 78350 Jouy-en-Josas, France; 2 SYSAAF Section Aquacole, Campus de Beaulieu, 35000 Rennes, France; 3 GABI, SIGENAE, INRA, AgroParisTech, Université Paris-Saclay, 78350 Jouy-en-Josas, France; 4 GeT-PlaGe, Genotoul, INRA US1426, 31320 Castanet-Tolosan Cedex, France; 5 Virologie et Immunologie Moléculaires, INRA, Université Paris-Saclay, 78350 Jouy-en-Josas, France; 6 Present Address: BIOEPAR, INRA, Oniris, Université Bretagne Loire, 44307 Nantes, France

## Abstract

**Background:**

Bacterial cold-water disease, which is caused by *Flavobacterium psychrophilum*, is one of the major diseases that affect rainbow trout (*Oncorhynchus mykiss*) and a primary concern for trout farming. Better knowledge of the genetic basis of resistance to *F. psychrophilum* would help to implement this trait in selection schemes and to investigate the immune mechanisms associated with resistance. Various studies have revealed that skin and mucus may contribute to response to infection. However, previous quantitative trait loci (QTL) studies were conducted by using injection as the route of infection. Immersion challenge, which is assumed to mimic natural infection by *F. psychrophilum* more closely, may reveal different defence mechanisms.

**Results:**

Two isogenic lines of rainbow trout with contrasting susceptibilities to *F. psychrophilum* were crossed to produce doubled haploid F2 progeny. Fish were infected with *F. psychrophilum* either by intramuscular injection (115 individuals) or by immersion (195 individuals), and genotyped for 9654 markers using RAD-sequencing. Fifteen QTL associated with resistance traits were detected and only three QTL were common between the injection and immersion. Using a model that accounted for epistatic interactions between QTL, two main types of interactions were revealed. A “compensation-like” effect was detected between several pairs of QTL for the two modes of infection. An “enhancing-like” interaction effect was detected between four pairs of QTL. Integration of the QTL results with results of a previous transcriptomic analysis of response to *F. psychrophilum* infection resulted in a list of potential candidate immune genes that belong to four relevant functional categories (bacterial sensors, effectors of antibacterial immunity, inflammatory factors and interferon-stimulated genes).

**Conclusions:**

These results provide new insights into the genetic determinism of rainbow trout resistance to *F. psychrophilum* and confirm that some QTL with large effects are involved in this trait. For the first time, the role of epistatic interactions between resistance-associated QTL was evidenced. We found that the infection protocol used had an effect on the modulation of defence mechanisms and also identified relevant immune functional candidate genes.

**Electronic supplementary material:**

The online version of this article (10.1186/s12711-018-0431-9) contains supplementary material, which is available to authorized users.

## Background

Rainbow trout (*Oncorhynchus mykiss*) is a freshwater fish farmed in almost every continent, with production estimated at around 813,000 tons in 2014 (FAO). With the increase in production, resistance to diseases has become a major field of research in order to limit economic losses associated with diseases. *Flavobacterium psychrophilum* is the causative agent of bacterial cold-water disease (BCWD) also called rainbow trout fry syndrome in small fish [1, 2]. BCWD occurs worldwide and, according to [3], it is the second most important trout disease that affects French farms. It causes high mortalities (up to 70%) and deformities may occur in surviving fish [1, 2, 4], with important economic impacts. In spite of intensive research to develop efficient vaccines [5–7] and the recent commercialisation of a vaccine in some countries (ALPHA JECT ^®^ IPNV-Flavo 0.025 PHARMAQ), the usual way to combat the disease remains the use of antibiotic treatments, which raises environmental concerns and issues about the emergence of antibiotic resistance [8–10]. Therefore, there is a crucial need for other methods to control the disease. Selective breeding for natural genetic resistance to *F. psychrophilum* is a promising approach since previous studies have revealed the existence of genetic variation for this trait. Moderate heritabilities were estimated in European and North American domestic broodstocks [11–13], and Leeds et al. [14] demonstrated that genetic gain could be obtained in experimental conditions after two generations of selection. Quantitative trait loci (QTL) associated with resistance measured as time to death or survival have been detected using linkage or association studies [15–20] in which resistance to *F. psychrophilum* was assessed using injection protocols for experimental infection.

Madsen and Dalsgaard [21] and Garcia et al. [22] compared injection (intraperitoneal or intramuscular), immersion, immersion combined with stress (skin lesion or formalin treatment) and cohabitation with infected fish as infection challenge methods with *F. psychrophilum*. They concluded that the injection method was more reproducible than immersion and observed a higher mortality rate after injection challenge (70–90%), than after immersion challenge (30–55%). However, injection is a route of infection that bypasses the physical and immune barriers of skin and mucosa, and likely modifies the tissues that are targeted by the primary infection within the host. Similar differences between infection routes were also reported for other bacterial and viral pathogens that affect rainbow trout, such as *Yersinia ruckeri* [23], the infectious hematopoietic necrosis virus [24], and the viral haemorrhagic septicaemia virus [25]. Infection with *F. psychrophilum* seemed to be more efficient when fish were stressed by formalin treatment or when the skin was damaged prior to immersion [21, 22, 26, 27], which suggests that external barriers hinder entry of the bacteria. *F. psychrophilum* has been observed in the skin mucus, in gills, and in connective tissue of the fins and operculum of salmonid fish [28, 29], but the precise sites of entry of the pathogen remain unknown. Epithelia (skin, gills, nasopharynx and gut) are considered important portals of entry of pathogens [25, 30], even if the mucus layer constitutes an efficient barrier. Mucus contains multiple antimicrobial factors, such as lysozyme, proteins of the complement system, heat shock proteins or immunoglobulins, which are involved in specific or non-specific defence mechanisms [31–34]. In fact, many bacteria, both commensal and pathogenic, are commonly found in the mucus, and adhesion to mucus is a classical virulence factor [35, 36]. Composition of the skin microbiota, which plays a protective role against infection [37, 38], is partly under genetic control, and QTL that are associated with abundance of some bacterial genera that are known to provide protection against pathogens have been identified [37].

Immersion challenge with *F. psychrophilum* probably reflects natural infection of rainbow trout better than injection. Thus, we developed a reproducible immersion challenge that does not involve preliminary stress [39] and used it to investigate the genetic variation of trout resistance to *F. psychrophilum*. Compared to injection challenge, immersion challenge may reveal QTL that drive defence mechanisms associated either with the entry of the bacterium into the host or with the host response once the pathogen has entered via the “natural” route, expanding the possibility of investigation of host antibacterial response. In this study, we took advantage of homozygous doubled haploid (DH) trout lines with contrasting susceptibilities to *F. psychrophilum* to search for resistance-associated QTL, using both routes of infection, as a first step towards a better understanding of the host response to infection and the identification of candidate (causative) genes. Investigating the resistance to *F. psychrophilum* is difficult since the establishment of reproducible experimental challenges, especially with immersion protocols, is very complicated. DH isogenic lines with contrasting resistance levels to *F. psychrophilum* represent a very useful resource to perform such experimental challenges and to identify susceptible versus resistant genetic backgrounds. Moreover, the use of DH lines allows powerful and simple genetic analyses [40, 41] with designs that are relevant to investigate interactions between QTL. Such interactions likely contribute to the variability of complex traits [42] but, to date, have been scarcely investigated.

## Methods

### Experimental QTL family

At INRA, we have established a collection of 16 isogenic homozygous rainbow trout lines that were derived from the INRA SY rainbow trout population after two generations of gynogenetic reproduction and further propagated by within-line single pair mating [43]. The lines have been screened for resistance to various diseases including several viruses [43–45] and more recently, for resistance to *F. psychrophilum*, using either injection or immersion as routes of infection [46, and unpublished results].

In this study, we selected two lines (B57 and AP2) with contrasting resistance to *F. psychrophilum* as F0 grandparents to produce the QTL family. Overall, line AP2 ranked among the most resistant of the 16 lines whereas B57 was consistently more susceptible (see Additional file 1: Figure S1).

One B57 female was mated to one AP2 sex-reversed male to produce a F1 isogenic family, consisting of all females that share the same genetic background and that are heterozygous at loci for which different alleles were fixed in the two F0 isogenic lines. One single F1 female was reproduced using mitotic gynogenesis in order to produce the QTL mapping family. Eggs were fertilized with UV-genetically inactivated milt and heat-shocked soon after fertilization in order to produce DH progeny by inhibition of the first embryonic mitosis [47]. Thus, the resulting offspring carried only one grandparental allelic variant at each locus. Males that were homozygous for a dominant body colour variant (golden phenotype) were used as milt donors for gynogenesis. The lack of golden fry in the progeny and of surviving fry in the haploid control (no heat-shock after fertilization with irradiated milt) served as control of the efficiency of the irradiation process.

Since DH progeny are homozygous, the power of QTL detection is increased by accurate evaluation of the effect of allelic substitution [40, 41, 48]. In the context of the recent whole-genome duplication event that occurred in the salmonid ancestor, DH individuals can also facilitate single nucleotide polymorphism (SNP) calling and genotyping in rainbow trout, and decrease the false discovery rate of paralogous sequence variants (PSV) as putative true allelic SNP variants [49].

F0 and F1 breeders were reared and spawned at the INRA PEIMA experimental farm (Sizun, France). The F2 progeny were incubated at the PEIMA farm. Eyed eggs were transported to the INRA IERP facilities (Jouy-en-Josas, France), iodine disinfected, and placed into rearing units that were supplied with recirculated, de-chlorinated tap water at a constant temperature of 10 °C. In total, 558 F2 progeny were produced, among which 372 were used for QTL detection (genotyping and phenotyping in infectious challenge). The 186 remaining un-phenotyped progeny were added to the genotype dataset in order to construct a more precise linkage map. At about three months post-hatching, the 372 QTL progeny were anaesthetized with 2-phenoxyethanol (0.2 mL/L), individually tagged (intraperitoneal implantation of a micro chip “Biolog-Tiny ID”) and reared under standard conditions (constant 10 °C and a commercial diet) until infectious challenges.

### Phenotyping for resistance to *F. psychrophilum*

Two different routes of infection, i.e. immersion and intramuscular injection, were compared. In both cases, fish were inoculated with *F. psychrophilum* FRGDSA 1882/11, a strain that was isolated in 2011 from a diseased rainbow trout during a severe outbreak in a trout farm in the South-West of France and belonging to the clonal complex CC-ST90 [50]. For both immersion and injection challenges, *F. psychrophilum* was grown at 18 °C in TYES broth on a rotatory shaker at 200 rpm until late exponential phase (OD_600_ approximately 1). Broth cultures were used for infection experiments following a posteriori bacteria counting by inoculation of serial dilutions on agar and counting of visible colonies after 48 h of incubation at 18 °C.

The immersion challenge was carried out when fish were about 5 months old, with a mean body weight of 4.7 ± 1.3 g. Prior to infection, 225 fish were randomly sampled from the QTL progeny and equally distributed into three 10-L aquaria. Each fish was weighed and its individual tag was recorded. After a few days of acclimation, fish were infected by immersion for 4 h in a bacterial suspension (approximately 8.10^7^ cfu/mL) in static water maintained at 10 °C with vigorous aeration. Bacteria were counted in water as above. Preliminary tests had revealed that *F. psychrophilum* strain FRGDSA 1882/11 is highly virulent when inoculated by injection and causes extremely high mortality in young fish. In order to fine-tune the level of the infectious dose and to be able to discriminate between susceptible and resistant fish for QTL detection, the injection challenge was performed when fish reached a larger size (around 8 months old with a mean body weight of 21.5 ± 6.9 g).

For the injection challenge, the broth culture was centrifuged and bacterial cells were rinsed once in saline buffer. Drops (25 µL) of serial dilutions of bacterial suspension were inoculated on agar for counting. As for immersion, 147 F2 progeny were randomly distributed into three 10-L aquaria (47–50 fish per aquarium). After a few days of acclimation, fish were anaesthetized and received an intramuscular injection of 50 µL of bacterial suspension, corresponding to approximately 145 CFU/fish, close to the dorsal fin.

After infection (immersion or injection), fish were kept at 10 °C with adequate water flow. Two fish that died within the first 2 days after infection were discarded (one for each challenge). Mortality was monitored twice a day. Dead fish were identified individually by tag recording. When mortality reached a plateau (49 and 35 days for the immersion and injection challenge, respectively), surviving fish were sacrificed by anaesthetic overdose, weighed and identified. A piece of caudal fin was clipped from all individuals at the time of identification and stored in 100% ethanol for DNA extraction. Two fish from the immersion challenge and three fish from the injection challenge were discarded because tag recording was not possible. For each challenge, the post-challenge dataset included body weight at the time of challenge, survival status of each fish (dead or alive at the end of the challenge) and time to death (in days after infection, for dead fish only).

### SNP genotyping

#### RAD sequencing and library preparation

In total, DNA was extracted from 555 fin samples using the Wizard Genomic DNA purification kit (Promega) with an RNAse step. Total DNA was quantified by measuring optical density at 260 nm (OD_260_) with a Qubit fluorometer. DNA quality was assessed by the OD_260_/OD_280_ ratio and by visual control on gel electrophoresis. All samples were diluted to 100 ng/µL before they were sent for RAD sequencing. Samples included the two F0 grandparents (AP2 and B57), the F1 female parent (and another isogenic F1 individual as backup) and 551 F2 DH progeny, among which the 365 progeny used for QTL detection (222 for immersion and 143 for injection, respectively) and the 186 additional F2 progeny that were genotyped only to strengthen SNP calling and the linkage map. To ensure sufficient sequencing depth of breeders, samples of the F0 and F1 parents were replicated (four replicates for F0 and eight replicates for F1). DNA samples were sent to the GeT-PlaGe sequencing platform ([51], Toulouse, France,) for restriction-site-associated DNA sequencing (RADseq) according to the protocol in [52]. Each DNA sample was digested with the *SbfI* restriction enzyme and then barcoded by adding P1 adaptors, which contained a 5-bp nucleotide barcode that differed by at least three nucleotides. Twelve sequencing libraries were generated with 48 pooled samples. Libraries were subsequently cut to a size of less than 800 bp by sonication. After size selection (250 bp on average) on agarose gel, the pooled libraries were purified, ligated to a P2 adaptor, and amplified by PCR. RAD libraries were paired-end sequenced (100 bp paired-end reads) on an Illumina HiSeq 2500 sequencer at the GeT-PlaGe sequencing platform.

#### Single nucleotide polymorphism discovery and genotyping

First, sequencing reads were demultiplexed and assigned to a single individual allowing no mismatch in the P1 barcode and one mismatch in the restriction site. Data from seven samples from F2 progeny that had less than one million reads were removed from the analysis. In order to facilitate detection of PSV [49], the dataset was supplemented with 20 DH individuals that were used as external controls. These DH individuals were sequenced in previous projects using the same RAD-sequencing methodology and restriction enzyme (unpublished data).

For SNP calling, sequence reads were processed through a de novo analysis using the *core* pipeline of the software Stacks version 1.19 [53, 54]. PCR duplicates were removed using the Stacks clone-filter program. Reads from replicates of the F0, F1 and F2 individuals were merged into a single sequence file per individual. In a first step, the ustacks program was used to identify putative loci for each sample. The minimum depth of coverage required to create a stack (group of identical reads or putative allele) was set at 3 (-m option), while the maximum distance allowed between stacks, was set at 2 nucleotides (-M option). These nucleotide differences take potential SNP and sequencing errors into account. Only primary reads were used (-N option equal to 0). The maximum number of stacks (i.e. maximum number of alleles) at a single de novo locus was set at 2 (max_locus_stacks option) in order to allow detection of putative duplicated loci. Next, the cstacks program was used to create a catalogue of loci by setting the number of mismatches allowed between samples to 1 (-n option). Finally, genotypes of all individuals (F0, F1, F2 and DH controls) were called using the sstacks program that matches individual stacks against the catalogue. The Stacks population program was used to calculate the frequency of genotypes at each locus.

#### Quality control of SNPs and removal of duplicated loci

Sequence data from the two F1 individuals were used to identify polymorphic and monomorphic loci. To be consistent with the pedigree of the QTL family (DH progeny of a cross between two DH homozygous grandparents), only bi-allelic loci were considered (17,460 out of 20,305 polymorphic loci identified in the Stacks catalogue).

*DH control population* The two F0 grandparents and the 20 external DH individuals served as controls to detect putative duplicated loci. Since all DH individuals are expected to be homozygous, a heterozygous genotype at a given locus indicates a PSV rather than a true allelic variant. Therefore, all loci that appeared heterozygous in at least two DH control individuals were discarded (list provided in Additional file 2: Table S1).

*Filtering of loci and individuals* Sequence data from 37 individuals were removed because of technical problems. Filtering of loci and individuals was based on call rate and call frequency as follows: F2 individuals with genotype calls for less than 20% of genotyped loci and loci with genotype calls for less than 70% of F2 individuals were discarded. Homozygosity for each F2 individual was checked on the 11,570 remaining loci and 24 fish were found to have a heterozygosity rate higher than 1%. Previous studies performed on isogenic lines [45, 49] assumed that residual heterozygous loci may be due to unreduced ova, donor milt contamination, mutation or sequencing errors. We did not find any F2 individual that was heterozygous at loci where the two grandparents shared the same allele, so contamination by donor milt was excluded. Spontaneous retention of a second polar body is suspected to be at the origin of unreduced ova. Under this hypothesis, a number of loci will retain the heterozygosity of the mother, at a frequency that depends on recombination during meiosis [40, 55]. Since recombination rates are higher in telomeric than centrometric regions of chromosomes [56, 57], a higher residual heterozygosity level is expected in telomeric regions. For the 24 fish that had an overall heterozygous rate higher than 1% (min: 1.12%; max: 65.04%), a gradient of heterozygosity rate was observed along the chromosome arms, which was consistent with the proportion of heterozygous loci being higher in telomeric regions than around the centromere (see Additional file 3: Figure S2). This supports the hypothesis that the 24 F2 progeny that exhibited a high frequency of heterozygous loci probably originated from spontaneously unreduced ova. Those individuals were discarded from the analysis. The remaining F2 fish had a heterozygosity rate lower than 1% and were considered as true doubled haploids. The remaining heterozygous loci were considered to result from sequencing errors or mutations and were set to missing genotypes before the last filtering step based on minor allele frequency (MAF) and individual coverage. Since the F2 fish were produced by mitotic gynogenesis from a single F1 female, a 1:1 ratio was expected for the alternative F0 alleles (AP2 and B57). Therefore, the remaining loci with a MAF lower than 0.30 were filtered out. In the end, only fish with more than 90% of called genotypes were kept for further analysis.

### Construction of the linkage map

The 1.2 version of the CarthaGène software [58, 59] was used to build the F2 family linkage map. The DH progeny were described as a backcross in the CarthaGène software. The group command was used, with the thresholds for two-point distance (Haldane/Ray) and logarithm of odds (LOD) set at 0.3 and 15, respectively. Linkage groups were assigned to trout chromosomes by blasting the sequences of RAD markers on the most recent published reference trout genome, Omyk_1.0 [60].

### QTL detection

QTL mapping was performed by chromosome with the QTLMap software [61] (version 0.9.8). For each chromosome, the hypothesis that one QTL (H1) versus no QTL (H0) affects the trait of interest was tested with the interval mapping method described in [62], using an approximate likelihood ratio test (LRT [63]) and scanning the chromosome in intervals of 1 cM. To take into account the fact that the F2 individuals are gynogenetic doubled haploids, they were coded as half-sib from a single sire (F1) and an unknown virtual dam that was different for each F2 fish [41]. The two challenges, injection and immersion, were analysed separately. The effects of body weight at challenge and aquarium on survival were tested with the average-to-average method (ANOVA) model on time to death and the logistic regression on the status as the end of the challenge with the R software, version 3.4.2 [64]. The effect of body weight was never significant but the aquarium effect was significant in all cases.

Using a model $M1$ that included aquarium as the only fixed effect, RESISTANCE was assessed using the unitrait Cox model option (calcul = 7; [61]) in QTLMap. Cox model fits a survival analysis model that makes no assumption on the trait distribution and that takes time to death and censoring into account [65, 66]. Surviving fish at the end of the period of survey corresponded to ‘censored’ observations, *i.e.* the expected event (death) was not observed during the observation period. In this analysis, the QTL effect is calculated for each genotype (allele) as a relative risk, with the B57 line origin taken as the reference (relative risk = 1). STATUS (dead/alive) was also analysed as a trait of practical interest for breeders, using the unitrait discrete distribution option of QTLMap (calcul = 2; [61]) with value 1 for survivors and 0 for dead fish. Using the variable STATUS, it was also possible to refine the model to search for additional QTL that might have been masked by effects of the main QTL and/or by epistatic interactions between QTL. A new model ($M2$) was applied, in which aquarium and the QTL detected for STATUS with model $M1$ were considered as fixed effects, along with interactions between the QTL used as co-factor and the newly detected QTL. To control the false discovery rate due to multiple-testing, the *P* values associated with interactions were corrected with the Benjamini–Hochberg method (BH) [67] implemented in the R software, version 3.4.2 [64] (stat package version 3.6.0, option p.adjust). Finally, ENDURANCE, which was defined as the time until death for fish that died during the experimental period [68], was investigated as a complementary description of possible host–pathogen interaction during infection. ENDURANCE was measured in dead fish only and was analysed using the unitrait Gaussian distribution option of QTLMap with model $M1$.

For each chromosome, when a QTL was suspected, the empirical distribution of the LRT was obtained with QTLMap from 1000 simulations (for STATUS and ENDURANCE) or permutations (for RESISTANCE) under the null hypothesis with trait heritability fixed at 0.5 for each chromosome. Then, we estimated the type-I error rejection threshold of H0 at the chromosome-wide level using the method described by Harrell and Davis [69]. A QTL with a chromosome-wide *P* ≤ 0.01 was considered significant. For each QTL that was chromosome-wide significant at *P* ≤ 0.001, the genome-wide level significance threshold was tested with 10,000 simulations/permutations under H0 and a Bonferroni correction to adjust the type-I error for number of chromosomes. Under H1, the QTL effect was estimated as the allelic substitution effect in a standard F2 progeny. Because progeny were doubled haploids, this effect corresponds to half the difference between the mean values of the trait in the two alternative homozygous progeny. The 95% confidence intervals (95% CI) of the QTL were obtained using the method of Li [70], in which the distribution of QTL locations is approximated from likelihood. For each significant QTL and each trait, we identified the grandparental origin of the allele at the QTL position, and thus determined the line origin of resistance/susceptibility. For all traits and each QTL, the maximum likelihood ratio test (LRTmax) curves were inspected visually. When the LRTmax curves showed two peaks, the hypothesis of two QTL (H2) versus one QTL (H1) was tested. Because the test was available only for the unitrait model for the time to death, fish that survived were given a time of death at d + 1, with d the day of death of the last fish that died, but it never reached the chromosome-wide significance level.

The percentage of phenotypic variance explained by a QTL in the DH progeny was calculated with the R software [64], using ANOVA for ENDURANCE and logistic regression for STATUS.

## Results

### Average performance after immersion and injection infectious challenges

**Fig. 1:**
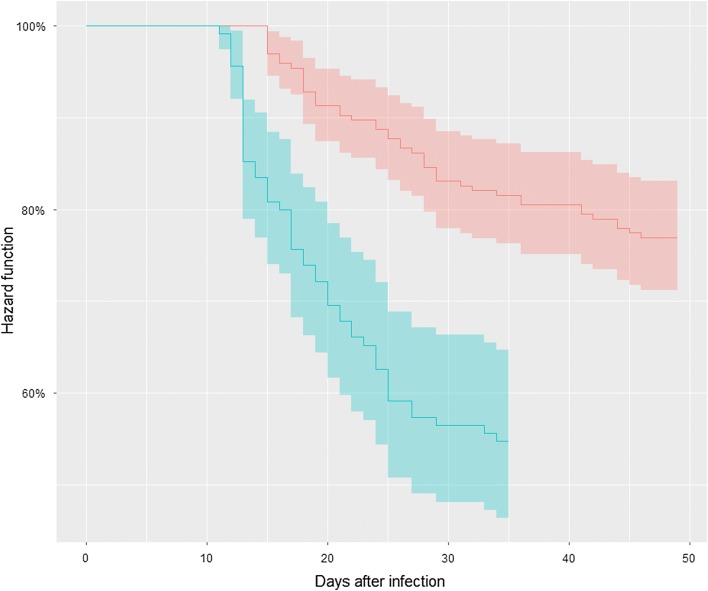
Survival curves after *Flavobacterium psychrophilum* infection of DH fish used for QTL detection. Kaplan–Meier estimation of survival functions after infectious challenges for the QTL family. The pink curve corresponds to the hazard function of DH progeny challenged by immersion (225 fish, 3 aquaria, 49 days) and the blue curve corresponds to the Hazard function of DH progeny challenge by intramuscular injection (147 fish, 3 aquaria, 35 days)

Fish from the QTL family were challenged with *F. psychrophilum* via two modes of infection: after the immersion challenge, the overall survival rate was 77% at day 48 (out of 225 infected fish) whereas after the injection challenge, it was 55% at day 34 (out of 147 infected fish) (Fig. 1). These values were in the range of survival rates recorded in previous challenges with the *F. psychrophilum* strain FRGDSA 1882/11. Regardless of the route of infection, individual body weight at the time of infection had no significant effect on time to death or on the final survival rate (STATUS). Fish that survived and died following the immersion protocol had the same mean body weight at the time of challenge (4.6 g). Accordingly, the mean body weight at the time of infection using injection was 20.9 g and 22.6 g (not significant) for surviving and dead fish, respectively (see Additional file 2: Table S2).

### Genotypes calls and linkage map

The catalogue of polymorphic loci, established with 511 individuals (507 unique F2’s, 2 F1’s and 2 F0’s), contained 17,460 bi-allelic loci of which 2867 were discarded from further analysis since they were putative duplicated loci (i.e. they were heterozygous in at least two DH controls, see list in Additional file 2: Table S1). After removing fish with a genotype call rate lower than 20% (n = 444) and markers with more than 70% missing genotypes (n = 11,570), 24 additional individuals that exhibited a rate of heterozygous loci higher than 1% were removed before the last filtering step based on MAF (> 0.30) and marker call rate (> 90%). The final dataset was composed of 9715 polymorphic loci and 427 F2 progeny (including 310 QTL progeny that were challenged with *F. psychrophilum* and 117 individuals with no phenotypic record). Of the 9715 markers, 9654 were mapped to 30 linkage groups (see Additional file 2: Table S3). The total length of the linkage map was 2645.2 cM. Linkage groups were successfully assigned to chromosomes using the genome assembly Omyk_1.0 [60]. As previously described [71, 72], chromosome 25 (Omy25) is separated into two chromosomes in the INRA SY population. In this paper, Omy25a corresponds to the short arm of Omy25 and Omy25b to its long arm. The 9654 markers accounted for 2130 distinct positions on the genetic map (see Additional file 2: Table S4). At each position, only the marker with the best call rate was kept for further QTL detection (see Additional file 2: Table S3). The final dataset for QTL detection included 310 F2 progeny, one F1 and the two F0 individuals, and 2130 markers.

### QTL detected following injection challenge

Using the $M1$ model, survival analysis with the Cox model revealed two genome-wide significant QTL associated with RESISTANCE after the injection challenge (*P* ≤ 0.005 at the genome-wide level) on Omy3 and Omy29 (named Omy3-QTL and Omy29-QTL, respectively). Two other QTL were chromosome-wide significant (*P* ≤ 0.01) on Omy10 and Omy26. For all QTL, the risk ratio was less than 1 (0.22–0.38, Table 1), which indicates that the allele for resistance originated from the AP2 (resistant) grandparent. The final survival rate ranged from 67 to 76% for individuals that were homozygous for the resistance (AP2) allele at all four QTL versus 34 to 50% for individuals that were homozygous for the susceptibility (B57) allele. Omy3-QTL and Omy29-QTL had the strongest effects (see Table 1). For STATUS (analysed as a binary trait), one chromosome-wide significant QTL was found on Omy25a and two genome-wide significant QTL on Omy3 and Omy29. These two QTL were the same as those detected for RESISTANCE (same location, same favourable (resistance) allele transmitted by AP2). They explained 14 and 12% of the phenotypic variation of the trait in the DH progeny, respectively, whereas Omy25a-QTL explained 7% of the phenotypic variation. Strikingly for this QTL, the susceptible grandparent (B57) transmitted the favourable allele. Likelihood ratio thresholds and flanking markers at each QTL are in Table S5 (see Additional file 2: Table S5). Figure S3 (see Additional file 4: Figure S3) presents the likelihood ratio profiles for each chromosome (1 cM interval) for the two resistance traits after the two types of infectious challenges.

**Table 1:** Results of QTL analyses for resistance traits after an injection challenge with *F. psychrophilum*

Trait	QTL	LRTmax	Position (cM)	CI (95%)	QTL effect	Resistance origin	Survival rate (%) according to allele origin at the QTL		% of phenotypic variance explained in DH progeny
							AP2	B57	
RESISTANCE	Omy3	21.24**	89	67–97	0.25	AP2	76	34	–
	Omy10	10.31*	23	4–93	0.35	AP2	67	50	–
	Omy26	9.49*	21	1–41	0.38	AP2	73	40	–
	Omy29	23.94***	48	26–49	0.22	AP2	76	38	–
STATUS	Omy3	21.28***	89	68–95	+ 0.29	AP2	76	34	14%
	Omy25a	11.06*	14	0–27	− 0.21	B57	49	60	7%
	Omy29	19.06***	48	25–49	+ 0.27	AP2	76	38	12%
ENDURANCE	Omy15	10.99*	11	0–78	+ 2.63	AP2	–	–	nc
	Omy29	9.90*	43	18–49	+ 2.63	AP2	–	–	11%

RESISTANCE: overall resistance, analysed with a Cox model survival analysis that takes failure, time to death and final survival (censored observations) into account; STATUS: (dead/surviving) phenotype at the end of the challenge, analysed as a binary trait; ENDURANCE: time to death in days after infection for dead fish only, analysed as a Gaussian trait; LRTmax = maximum of likelihood ratio test; Position in the genetic map in centimorgans (cM); CI = confidence interval; Chromosome-wide significant at *P ≤ 0.01; Genome-wide significant at **P ≤ 0.05 or ***P ≤ 0.01; The QTL effect was measured as the relative risk for RESISTANCE (B57 as the reference, risk = 1), as half the difference between the mean values of the two classes of homozygous progeny (individual values fixed as 1 for survivors and 0 for dead fish) for STATUS and as half the difference (in days) between the mean date of death of the two classes of homozygous progeny for ENDURANCE

For ENDURANCE, which was measured as time to death (hence, using only fish that died), two chromosome-wide significant QTL were detected on Omy15 and Omy29. Omy29-QTL explained 11% of the phenotypic variance of the trait in the DH progeny. Omy15-QTL is a new QTL, whereas Omy29-QTL is likely the same as that detected for RESISTANCE (close location, overlapping 95% confidence intervals, one flanking marker in common). For both QTL, the favourable allele originated from the resistant grandparent (AP2), and fish that carried the favourable allele at both QTL died on average 5 days later than fish with the unfavourable allele.

### QTL detected following immersion challenge

For the immersion challenge and with model $M1$, survival analysis with the Cox model revealed three QTL associated with RESISTANCE (Table 2). Two new chromosome-wide significant QTL located on Omy2 and Omy21, respectively. One genome-wide significant QTL on Omy3 matched the QTL that was detected in the injection challenge analysis (similar position, *i.e.* 88 and 89 cM for the immersion and injection challenge analyses, respectively, and resistance transmitted by AP2 in both cases). The risk ratios of the two newly detected QTL (3.73 for Omy2-QTL and 3.08 for Omy21-QTL, respectively) indicated that the favourable (resistance) allele originated from the susceptible grandparent (B57). Omy3-QTL had the largest effect, with 94 versus 53% survival for individuals that were homozygous for the AP2 and B57 allele, respectively. For Omy2-QTL and Omy21-QTL, the absolute difference in survival between alternative homozygotes was around 20% (Table 2). The QTL detected for STATUS (binary trait) provided results that are fully consistent with the results obtained for RESISTANCE. Omy3-QTL had the largest effect (explaining 18% of phenotypic variance in DH progeny), whereas Omy2-QTL and Omy21-QTL had the smallest effects (6 and 7% of phenotypic variance in DH progeny, respectively).

**Table 2:** Results of QTL analyses for resistance traits after an immersion challenge with *F. psychrophilum*

Trait	QTL	LRTmax	Position (cM)	CI (95%)	QTL effect	Resistance origin	Survival rate (%) according to allele origin at the QTL		% phenotypic variance explained in DH progeny
							AP2	B57	
RESISTANCE	Omy2	14.04*	14	3–32	3.73	B57	69	89	–
	Omy3	39.87***	88	82–93	0.09	AP2	94	53	–
	Omy21	12.65*	99	64–103	3.08	B57	66	87	–
STATUS	Omy2	13.17*	14	2–36	− 0.19	B57	69	89	6%
	Omy3	39.47***	88	81–93	+ 0.33	AP2	94	53	18%
	Omy21	12.36*	99	63–104	− 0.18	B57	66	87	7%
ENDURANCE	Omy20	12.54*	28	5–37	+ 4.93	B57	–	–	nc
	Omy27	13.56*	26	10–47	+ 5.65	B57	–	–	nc

RESISTANCE: overall resistance, analysed with a Cox model survival analysis that takes failure time to death and final survival (censored observations) into account; STATUS: (dead/surviving) phenotype at the end of the challenge, analysed as a binary trait; ENDURANCE: time to death in days after infection for dead fish only, analysed as a Gaussian trait; LRTmax = maximum of likelihood ratio test; Position in the genetic map in centimorgans (cM); CI = confidence interval; Chromosome-wide significant at *P ≤ 0.01; Genome-wide significant at ***P ≤ 0.01; The QTL effect was measured as the relative risk for RESISTANCE (B57 as the reference, risk = 1), as half the difference between the mean values of the two classes of homozygous progeny (individual values fixed as 1 for survivors and 0 for dead fish) for STATUS and as half the difference (in days) between the mean date of death of the two classes of homozygous progeny for ENDURANCE

For ENDURANCE after the immersion challenge, two new chromosome-wide significant QTL were detected on Omy20 and Omy27. Fish that carried the B57 allele at the two ENDURANCE QTL survived longer (about 10–11 days more) than those that carried the AP2 allele. The immersion or injection challenge had no common QTL associated with ENDURANCE.

### Detection of additional QTL based on co-factor analyses

Since QTL may have been masked by the major QTL described above and/or by possible epistatic interactions between them, analyses were refined using STATUS as the resistance trait. Model $M2$ was used, in which QTL detection was computed with the effect of each chromosome-wide and genome-wide significant QTL detected for STATUS with model $M1$ being fixed as co-factors alternatively. As shown in Table 3, use of model $M2$ revealed five additional chromosome-wide or genome-wide significant QTL (absolute effects of QTL on survival rates are in Additional file 5: Figure S4).

**Table 3:** Results of QTL analysis using the model *M2* for resistance trait following injection or immersion challenges

Infection route	QTL	LRTmax	Position (cM)	CI (95%)	Increase in survival rate		Resistance origin		*P* value fixed effect	*P* value interaction
					Fixed_R (%)	Fixed_S (%)	Fixed_R	Fixed_S		
IMMERSION	Omy17_**Omy3**_	13.97*	61	0–92	38	7	AP2	AP2	***	NS
	Omy25a_**Omy3**_	10.41*	4	0–35	10	18	B57	B57	***	NS
*Type 1 interaction*										
INJECTION	^a^Omy3_**Omy29**_	15.27**	89	46–105	16	47	AP2	AP2	***	***
IMMERSION	^b^Omy2_**Omy3**_	15.35**	97	63–104	4	39	B57	B57	***	***
	^b^Omy3_**Omy21**_	40.73***	87	82–93	20	55	AP2	AP2	***	***
	^c^Omy3_**Omy2**_	35.66***	87	81–94	17	44	AP2	AP2	***	***
INJECTION	^a^Omy29.2_**Omy3**_	14.85*	23	8–49	5	48	B57	AP2	***	*
	Omy17_**Omy25a**_	15.85**	73	53–79	11	53	AP2	B57	***	***
IMMERSION	Omy7.2_**Omy21**_	11.48*	7	0–103	5	31	AP2	B57	***	***
*Type 2 interaction*										
INJECTION	^d^Omy25a_**Omy3**_	25.49***	14	10–18	53	16	B57	B57	***	*
	^d^Omy3_**Omy25a**_	35.35***	89	86–92	59	22	AP2	AP2	***	***
	Omy26_**Omy29**_	11.75*	18	0–34	30	26	AP2	AP2	***	***
INJECTION	Omy17_**Omy29**_	18.29***	74	58–92	47	11	AP2	B57	***	***
IMMERSION	Omy24_**Omy2**_	12.71*	4	0–19	20	1	B57	AP2	***	***
*Type 3 interaction*										
IMMERSION	Omy7.1_**Omy2**_	16.42**	61	32–87	19	19	B57	AP2	***	***

The table presents chromosome-wide or genome-wide significant QTL detected for STATUS using model $M2$; Reciprocal interactions could be tested only for QTL detected in the first STATUS analysis (model $M1$); LRTmax = maximum of likelihood ratio test; Position in the genetic map in centimorgans (cM); CI = confidence interval; Chromosome-wide significant = *P ≤ 0.01; Genome-wide significant = **P ≤ 0.05 or ***P ≤ 0.01; *P* values for fixed effect and interaction corrected with Benjamini–Hochberg method: Non-significant = NS; *P value ≤ 0.05; ***P value ≤ 0.001

^a^The reciprocal interaction could not be tested as a new QTL (Omy29.2_**Omy3**_-QTL) was detected with the reciprocal model

^b,d^Reciprocal models for QTL pairs

^c^The QTL in the reciprocal model (Omy2_**Omy3**_-QTL) was only suggestive (*P* ≤ 0.05) at the chromosome wide level

In the analysis of the injection challenge, including the effect of Omy3-QTL as co-factor in model $M2$ allowed identification of an additional QTL on Omy29 (named Omy29.2_**Omy3**_-QTL). This Omy29.2_**Omy3**_-QTL was located at 23 cM, just at the limit of the confidence interval of the first QTL on Omy29 detected for RESISTANCE and STATUS with model $M1$ (see Table 1) for the injection challenge. The hypothesis that there were two QTL for STATUS was tested by fitting both Omy3-QTL and Omy29-QTL in the model; Omy29.2_**Omy3**_-QTL remained chromosome-wide significant (*P* < 0.01), which supports the existence of two different QTL on Omy29. Including Omy29-QTL in the model allowed the role of Omy26-QTL to be extended to STATUS (previously chromosome-wide significant for RESISTANCE only). Finally, new QTL were detected on Omy17 by fitting Omy25a-QTL or Omy29-QTL, respectively. Because Omy17_**Omy25a**_-QTL and Omy17_**Omy29**_-QTL were very close to each other, they were considered as a single QTL.

For the immersion challenge, fitting the effect of the Omy2-QTL revealed two new QTL (Omy7.1_**Omy2**_-QTL and Omy24_**Omy2**_-QTL). Fitting the effect of Omy3-QTL revealed a new QTL on Omy17 (Omy17_**Omy3**_-QTL) and a QTL on Omy25a (Omy25a_**Omy3**_-QTL). Interestingly these two QTL were detected on the same two chromosomes in the injection challenge analysis. Although their positions in each challenge were distinct, confidence intervals overlapped largely. Hence, we favour the hypothesis of a single QTL on each of these chromosomes. Taken together, these results support the idea that the functions encoded by the genes represented by the QTL on Omy17 and Omy25a could play a role in resistance regardless of the route of infection. Finally, fitting Omy21-QTL revealed another QTL on Omy7 (Omy7.2_**Omy21**_-QTL). This QTL was considered as distinct from Omy7.1_**Omy2**_-QTL since it was located far away (7 vs. 61 cM) and outside its confidence interval.

### Evidence for interactions between resistance-associated QTL

Most of the newly detected QTL defined above were detected only after the interactions between QTL were taken into account in the analysis with model $M2$. As shown in Table 3, interactions were suggestive (*P* ≤ 0.05) for two pairs of QTL and highly significant (*P* ≤ 0.001) for 11 other pairs of QTL. Since both grandparents (AP2 and B57) can transmit the allele for resistance depending on the QTL, the resistance/susceptibility alleles at QTL will be referred to as the R/S allele for the discussion of interactions between QTL, irrespective of their AP2 or B57 origin. The detected interactions could be classified into two main types and a third type that contained only one pair of epistatic QTL, as described in the following.

#### Type1 interaction: interacting QTL alternatively contribute to resistance

The first type of interaction, which was recorded for six pairs of QTL, was associated with a larger effect of one QTL when the other QTL was fixed at the S allele (see Table 3 for details). For these six pairs of epistatic QTL, the survival rate was significantly lower when both QTL carried the S allele than when at least one QTL carried the R allele (see Additional file 5: Figure S4). Therefore, each QTL alternatively contributes to resistance, depending on the allelic status at the other QTL. A typical case for such interaction is the Omy21_Omy3 QTL pair in the immersion challenge. Indeed, when one QTL was fixed at the S allele and the other QTL changed from S to R, the survival rate increased by 39 and 55%, respectively for Omy21_**Omy3_S**_-QTL and Omy3_**Omy21_S**_-QTL. In contrast, when one QTL was fixed at R allele, changing the allele of the other QTL from S to R resulted in an increase in survival of only of 4 and 20%, respectively. An intriguing feature was that for three epistatic QTL, Omy29.2_**Omy3**_-QTL and Omy17_**Omy25a**_-QTL in the injection challenge and Omy7.2_**Omy21**_-QTL in the immersion challenge, the origin of the favourable allele changed depending on the allele fixed for the QTL used as co-factor. For instance, origin of the favourable allele at Omy7.2_**Omy21**_-QTL was AP2 or B57, depending on the allele at the QTL on Omy21 (R or S).

#### Type 2 interaction: resistance at one QTL enhances the effect of the other QTL

The second type of interaction, detected for four pairs of QTL, resulted in a larger increase in survival rate when one of the two QTL was fixed at the R allele (see Table 3 for details). This interaction can be illustrated by the significantly greater survival rate when both QTL of a pair carried the R allele compared to any other combination of alleles (see Additional file 5: Figure S4).

The Omy3_Omy25a QTL pair in the injection challenge is an example of such an interaction, with an absolute increase in survival rate by 53 and 59%, respectively, when Omy25a_**Omy3_R**_-QTL and Omy3_**Omy25a_R**_-QTL changed from S to R. For Omy17_**Omy29**_-QTL in the injection challenge and Omy24_**Omy2**_-QTL in the immersion challenge, the large increase in survival rate was combined with an inversion of the origin of the favourable allele at the QTL when the QTL fitted in the model $M2$ carried the R versus the S allele.

#### Type 3 interaction: inversion of origin of the favourable allele

In the immersion challenge, the absolute effect of Omy7.1_**Omy2**_-QTL on survival did not depend on the allele at Omy2-QTL (+ 19%) but the origin of the favourable allele changed from B57 to AP2 depending on the R/S allele at Omy2-QTL.

### Key immune genes induced by *F. psychrophilum* infection co-located with resistance-associated QTL

In a previous study [46], we analysed the transcriptome response to *F. psychrophilum* in the pronephros of two trout isogenic lines (B57 and A3) with contrasting susceptibilities to *F. psychrophilum*, using micro-arrays. A list of 571 differentially-expressed genes after *F. psychrophilum* injection in at least one of these lines was generated ([46] and supplementary material in [73, 74]). All probes corresponding to these genes were mapped on the rainbow trout genome [60], to test whether the differentially-expressed genes are located close to a QTL. Probe positions were compared to the rainbow trout annotation [75] to name the corresponding proteins. Sixty-four probes (corresponding to 49 genes) were located within or close to the 95% confidence intervals of the QTL detected in the current study (see Additional file 2: Tables S5 and S6). Among these 49 genes, 14 had functions that suggest that they may be involved in the resistance controlled by the respective QTL. These genes can be classified into four functional categories: (1) bacterial sensors and damage associated molecular pattern (DAMP) molecules (*cd209* and other *c*-*type Lectin*-*4*, *tlr2*; and *hmgb3*); (2) inflammatory factors (*steap4*; *il1r2*; and *drtp1*); (3) effectors killing bacteria (*c3*; *hamp*) or affecting the host (*collagenase/mmp13*); and (4) interferon stimulated genes (ISG) (*vig2*, *ifi44*, and *ifitm*). A detailed description of these genes and their function is in Additional file 6.

## Discussion

In this study, we investigated the genetic architecture of resistance to *Flavobacterium psychrophilum* in rainbow trout using a cross between two isogenic grandparental lines with contrasting susceptibilities to the bacterium. Two different infection modes, immersion and injection were used to detect QTL that were in segregation in the two grandparent isogenic lines. Although injection is commonly used in protocols of experimental infection with *F psychrophilum*, immersion is more relevant since it likely mimics the natural infection more closely. However, reliable and reproducible immersion challenges are more difficult to establish than injection challenges, especially if a large number of fish are to be infected. Using a DH QTL family produced from isogenic lines with well-established susceptibility was a unique way to facilitate the genetic analysis. Interestingly, QTL detected in both infection models overlapped partly, which supports the hypothesis that a core set of immune mechanisms is recruited, while others can be specific to the infection route. This study also provides the first evidence of epistasis among QTL associated with resistance to *F. psychrophilum*. Finally, we also investigated endurance of the fish (*i.e.,* time to death after infection) and detected four QTL associated with this trait. It should be noted that only one endurance QTL was also defined as a resistance QTL (Omy29-QTL), which indicates that these two traits are partly driven by different mechanisms.

Altogether, we detected 12 QTL associated with resistance to *F. psychrophilum* (see Table 4 for a summary). Three QTL were common to both routes of infection (Omy3-QTL, Omy17-QTL and Omy25a-QTL), four were detected after injection only (Omy10-QTL, Omy26-QTL, Omy29-QTL and Omy29.2-QTL) and five after immersion only (Omy2-QTL, Omy7.1-QTL, Omy7.2-QTL, Omy21-QTL and Omy24-QTL). Three of these QTL (Omy21-QTL, Omy29-QTL and Omy29.2-QTL) had not been detected in previously published studies [16, 18, 19, 49]. Some QTL could be detected only after taking the effect of—or the interaction with—another QTL with strong effect into account. Among the QTL that govern resistance for both types of challenge, Omy3-QTL was the most significant and explained 14 and 18% of the phenotypic variation in survival rate of the DH progeny following injection and immersion, respectively.

**Table 4:** Summary of all QTL associated with resistance and endurance

QTL	Injection		Immersion		QTL found on the same chromosome in
	Resistance	Endurance	Resistance	Endurance	
Omy2			*M1*		[16, 18]
Omy3	*M1*		*M1*		[20]
Omy7.1			*M2*		[16, 19]
Omy7.2			*M2*		[20]
Omy10	*M1*				[20]
Omy15		*M1*			[18]
Omy17	*M2*		*M2* ^*a*^		[16]
Omy20				*M1*	[16]
Omy21			*M1*		
Omy24			*M2*		[16]
Omy25a	*M1*		*M2* ^*a*^		[20]
Omy26	*M1*				[18]
Omy27				*M1*	
Omy29	*M1*	*M1*			
Omy29.2	*M2*				

In this table, the term “Resistance” combines the QTL detected for STATUS and/or RESISTANCE traits

*M1*: QTL detected using the *M1* model, *M2*: QTL detected using the *M2* model with fixed effect and interactions, M2^a^ interaction non-significant or suggestive (5%)

We identified several QTL that seem specific to a given route of infection. One cannot exclude that these QTL contribute to resistance regardless of the mode of infection but that they were not detected in one challenge because of lack of power of the experiment. However, the observation of QTL specific to the route of infection is consistent with our unpublished results, that show a moderate genetic correlation between the survival of standard trout families after immersion or injection challenge. The five QTL that were detected only after immersion could drive protective mechanisms that are related to entry of the bacterium into the host at the skin or mucosa level, or mechanisms that would be triggered significantly only when the bacterium has entered the host after an immersion challenge. For example, resident phagocytes that are located close to the « natural » entry sites could mediate such mechanisms. These cells can sense the pathogens, become activated, mediate a local innate response and/or migrate to the spleen or to other sites where the immune responses take place. In contrast, QTL detected only after injection could correspond to resistance mechanisms that become more critical when the first line of natural defence has been by-passed.

Age of the fish at the time of immersion and injection challenges may also have influenced the relative effect of the QTL. Because the *F. psychrophilum* strain used is highly virulent, it can induce very high mortality rates when injected to small fish even at low doses, which may prevent detection of QTL. Hence, we chose to carry out the injection challenge on 8-month-old fish, while the immersion challenge was performed on younger fish (5-month-old, to better simulate the natural infection at early stages), having a potentially more immature immune system. Importantly, 5-month-old fish already have a rather mature immune system. For example, they already have B and T lymphocytes, and can mount adaptive immune responses [76]. The same study showed that a strong transcriptional response to bacterial infection can be detected since the eyed egg stage. While drastic changes of this response were observed before the first feeding stage, fewer changes occur from first feeding and 3 weeks later. Thus, age-dependent changes in immune response that take place in trout after several months of independent feeding life correspond to maturation of an established immune system, rather than to a dramatic change, and we do not expect drastic differences in immune responses for 5- versus 8-month-old fish. Moreover, our experience with isogenic lines indicates that lines that are highly susceptible to *F. psychrophilum* infection remain susceptible during their first year of life and often throughout life (unpublished). Along the same line, the general conclusion of comparisons of susceptible versus resistant lines across a range of sizes is that lines rank consistently regarding susceptibility to *F. psychrophilum* ([46] and unpublished results), supporting the hypothesis that maturation of the immune system does not lead to drastic changes in the susceptibility/resistance status of the fish in this study. However, some pathways that are critical at young stages may become less critical as adaptive defence mechanisms get more effective at later ages.

To get further insight into the mechanisms involved in host response, we combined the positional information obtained from QTL mapping with results of our previous functional study. This pointed to several key genes involved in antibacterial immunity, which are induced in the pronephros of fish injected with *F. psychrophilum*, and are located in the vicinity of the resistance QTL. These genes are potentially interesting for the validation of a repertoire of candidate genes in more restricted QTL regions.

Few studies have addressed interactions between QTL, although it has been shown that epistasis may play an important role in the genetic variation of complex traits [42, 77] and that taking epistasis into account allows more QTL to be detected [42, 78, 79]. In the current study, five of 15 QTL were detected only after epistasis was taken into account, which provides further insights into the genetic architecture of resistance to *F. psychrophilum* and the complexity of the underlying mechanisms.

Two major types of interactions were identified. In the first type, the effect of each QTL was present or enhanced only when the other QTL was fixed at the susceptibility allele. Consequently, each QTL alternatively contributes to the resistance (“compensation-like” effect). This phenomenon occurred between the three most significant immersion QTL and between two of the most significant QTL that were identified following injection. In particular, “compensation-like” interaction was detected between Omy2-QTL and Omy3-QTL that carry candidate genes coding for anti (*steap4*) and pro (*il1r2*) inflammatory factors. The second type of interaction results in an “enhancing-like” effect of the resistance allele of one QTL on the effect of other QTL, resulting in a gain in resistance only when both QTL carry their resistance allele. Therefore, mechanisms associated with the R alleles at the two QTL may be synergetic or, alternatively, the presence of the S allele at one QTL may hinder the efficiency of the resistance mechanisms encoded at the other. This interaction was detected between Omy29-QTL and two other QTL (Omy26-QTL and Omy17-QTL). In other cases, as for the Omy2_Omy7.1 pair of QTL or in some cases among type 1 and 2 interactions, there was what may be called a “counter-acting interaction” with an inversion of the favourable allele at one QTL when switching the allele at the alternative QTL. Such a type of interaction was described in studies on lung [80] or colon [81] cancer in recombinant congenic strains of mouse for which QTL were expected to be important components of tumour susceptibility. Further investigations are needed in order to better understand the underlying immune pathways, which might correspond to negative feedback loops.

Several QTL associated with resistance to *F. psychrophilum* had a strong effect on resistance in the QTL family used in this study. Among those, the QTL on Omy3 is of particular interest because it controls resistance following both routes of infection. This QTL has also been detected in two American trout populations [20] and might be relevant in other populations. Our study also confirms the role of several other QTL that were previously detected in American trout populations in response to infection with a different strain of *F. psychrophilum* (CSF-259-93) [16, 18–20]. Hence, these QTL are not specific to a particular strain of *F. psychrophilum* nor to a particular host population, which strengthens their potential interest for breeding purposes. It is noteworthy that the FRGDSA 1882/11 and CSF-259-93 strains both belong to the same mPCR type 2, which is one of the major types identified in *F. psychrophilum* isolates from worldwide origins [50, 82].

Our results also suggested that a given type of infection challenge (route of infection and/or age of fish) may select for specific resistance mechanisms that may not be as relevant in other contexts. The challenge model used to select candidates may result in gene-environment interactions with less genetic progress than expected in the context of natural infection in farms. However, choosing the ‘best’ infection challenge for a selection programme should take into account not only genetic, but also practical and economic issues. From a practical point of view, an encouraging result of this study is that, within the range of fish size we investigated, individual body weight at the time of challenge had no or little effect on resistance, meaning that individual tagging to record fish weight may not be necessary when implementing an infection challenge.

## Conclusions

In this paper, we confirmed the complex genetic determinism of resistance to *Flavobacterium psychrophilum* in rainbow trout. Some QTL that drive a significant part of the phenotypic variance in different infectious contexts were detected and deserve further confirmation in standard trout families. Several genes involved in response to *F. psychrophilum* infection were associated with the detected QTL, providing a preliminary list of relevant candidate genes. Finally, this study highlighted the role of epistatic interactions between resistance QTL (and thus between the underlying mechanisms) and for the first time, evidenced the effect of the type of infection protocol with *F. psychrophilum* on the balance between different resistance mechanisms.

## Additional files

**Additional file 1: Figure S1.** Cumulative survival curves of fish from AP2 and B57 grandparental isogenic lines infected with *Flavobacterium psychrophilum.* Description: Fish were infected with the *F. psychrophilum* FRGDSA 1882/11 strain and mortality was recorded for 29 days post-infection. For injection protocol (—), 100 fish (average weight of 10.3 g for AP2 and 12.2 g for B57) were infected with (a): 450 CFU/mL (replicate of 50 fish) or (b): 300–550 CFU/mL (replicate of 50 fish). For the immersion protocol (- - - - -), 100 fish (average weight of 1.1 g for AP2 fish and 1.0 g for B57 fish) were infected by immersion for 4 h in a bacterial suspension (approximately 3.10^7^ CFU/mL) in static water maintained at 10 °C with vigorous aeration, in two replicates of 50 fish each (a) and (b).

**Additional file 2:** **Table S1.** List of putative duplicated loci: heterozygous RAD SNPs detected in at least two doubled haploid control individuals (DH controls), each from different rainbow trout isogenic lines. **Table S2.** Overall survival and weight of fish of the QTL family at the end of the immersion and injection infectious challenges. Description: Data were recorded for 49 and 35 days for the immersion and injection challenges, respectively. **Table S3.** Genetic linkage map constructed with the F2 progeny. Description: The marker at one position used in the QTL detection is shown in red bold. **Tables S4.** SNP allele and position for the 2130 markers used in the QTL detection. Description: SNP position is given in bp from the first bp of the read. **Table S5.** All significant (*P* ≤ 0.01 at the chromosome wide level) detected QTL, error-I rejection threshold at chromosome and genome-wide levels calculated for each QTL, flanking markers at each QTL, with name and position on the linkage map (cM). **Table S6.** List of 49 up- and down- regulated genes after *F. psychrophilum* infection in two rainbow trout isogenic lines in [46] located in the QTL associated with resistance traits identified in this study. **Table S7.** Reads of the 9654 polymorphic loci used in the linkage map and QTL detection.

**Additional file 3: Figure S2.** Evolution of the rate of hererozygosity along chromosomes (mean values for the 24 individuals with an overall rate of heterozygosity higher than 1%). Description: Metacentric chromosomes (a): under the hypothesis of spontaneous retention of the second polar body during meiosis, the rate of heterozygosity is expected to be lower around the centromere (—) than in telomeric regions (**- - - -**). Acrocentric chromosomes (b): under the hypothesis of spontaneous retention of the second polar body during meiosis, the rate of heterozygosity is expected to increase along the chromosome from the centromeric region to the telomere. Data are illustrated for 15 chromosomes.

**Additional file 4: Figure S3.** Graphical compilation of likelihood ratio profiles calculated for each chromosome (1-cM interval) for the two resistance traits after the two modes of infection challenges. Description: (a) RESISTANCE and (b) STATUS after injection challenge, (c) RESISTANCE and (d) STATUS after immersion challenge. For each chromosome, horizontal bars indicate the corresponding significance thresholds (green: *P* ≤ 0.01 at the chromosome-wide level; red: *P* ≤ 0.05 at the genome-wide level).

**Additional file 5: Figure S4.** Final survival rate according to the allele origin at pairs of epistatic QTL for resistance to infection with *F. psychrophilum*. Description: For each figure, abscissa corresponds to the combination of favourable (R) and unfavourable (S) alleles with the grandparent origin in colour (green for B57 and red for AP2) for each pair of epistatic QTL OmyA_OmyB. Survival rates (in ordinate) with similar letters are not significantly different (Fisher exact test *P* ≤ 0.05 and Benjamini–Hochberg correction for multiple testing of stat package from R software).

**Additional file 6.** Details on the immune genes induced by *F. psychrophilum* infection that map within resistance-associated QTL.

## References

[1] Nematollahi A, Decostere A, Pasmans F, Haesebrouck F (2003). *Flavobacterium psychrophilum* infection in salmonid fish. J Fish Dis.

[2] Starliper CE (2011). Bacterial coldwater disease of fishes caused by *Flavobacterium psychrophilum*. J Adv Res..

[3] Guichard B (2004). Principaux résultats de l’enquête « Pathologie de poissons 2004 ». Bull Epidemiol..

[4] Lorenzen E, Dalsgaard I, Bernardet JF (1997). Characterization of isolates of *Flavobacterium psychrophilum* associated with cold water disease or rainbow trout fry syndrome I: phenotypic and genomic studies. Dis Aquat Org..

[5] Fredriksen BN, Olsen RH, Furevik A, Souhoka RA, Gauthier D, Brudeseth B (2013). Efficacy of a divalent and a multivalent water-in-oil formulated vaccine against a highly virulent strain of *Flavobacterium psychrophilum* after intramuscular challenge of rainbow trout (*Oncorhynchus mykiss)*. Vaccine..

[6] Plant KP, LaPatra SE, Cain KD (2009). Vaccination of rainbow trout, *Oncorhynchus mykiss* (Walbaum), with recombinant and DNA vaccines produced to *Flavobacterium psychrophilum* heat shock proteins 60 and 70. J Fish Dis.

[7] Plant KP, LaPatra SE, Call DR, Cain KD (2014). Attempts at validating a recombinant *Flavobacterium psychrophilum* gliding motility protein N as a vaccine candidate in rainbow trout, *Oncorhynchus mykiss* (Walbaum) against bacterial cold-water disease. FEMS Microbiol Lett.

[8] Schmidt AS, Bruun MS, Dalsgaard I, Pedersen K, Larsen L (2000). Occurrence of antimicrobial resistance in fish-pathogenic and environmental bacteria associated with four Danish rainbow trout farms. Appl Environ Microbiol.

[9] Muziasaria WI, Pärnänen K, Johnson TA, Lyra C, Karkman A, Stedtfeld RD (2016). Aquaculture changes the profile of antibiotic resistance and mobile genetic element associated genes in Baltic Sea sediments. FEMS Microbiol Ecol..

[10] Duman M, Altun S, Cengiz M, Saticioglu IB, Buyukekiz AS, Sahinturk P (2017). Genotyping and antimicrobial resistance genes of *Yersinia ruckeri* isolates from rainbow trout farms. Dis Aquat Org..

[11] Henryon M, Jokumsen A, Berg P, Lund I, Pedersen PB, Olesen NJ (2002). Genetic variation for growth rate, feed conversion efficiency, and disease resistance exists within a farmed population of rainbow trout. Aquaculture.

[12] Henryon M, Berg P, Olesen NJ, Kjær TE, Slierendrecht WJ, Jokumsen A (2005). Selective breeding provides an approach to increase resistance of rainbow trout (*Onchorhychus mykiss*) to the diseases, enteric redmouth disease, rainbow trout fry syndrome and viral hemorrhagic septicemia. Aquaculture.

[13] Silverstein JT, Vallejo RL, Palti Y, Leeds TD, Rexroad CE, Welch TJ (2009). Rainbow trout resistance to bacterial coldwater disease is moderately heritable and is not adversely correlated with growth. J Anim Sci.

[14] Leeds TD, Silverstein JT, Weber GM, Vallejo RL, Palti Y, Rexroad CE (2010). Response to selection for bacterial cold water disease resistance in rainbow trout. J Anim Sci.

[15] Campbell NR, LaPatra SE, Overturf K, Towner R, Narum SR (2014). Association mapping of disease resistance traits in Rainbow trout using restriction site associated DNA sequencing. G3 (Bethesda)..

[16] Vallejo RL, Palti Y, Liu S, Evenhuis JP, Gao G, Rexroad CE (2014). Detection of QTL in Rainbow trout affecting survival when challenged with *Flavobacterium psychrophilum*. Mar Biotechnol (NY)..

[17] Vallejo RL, Palti Y, Liu S, Marancik DP, Wiens GD (2014). Validation of linked QTL for bacterial cold water disease resistance and spleen size on rainbow trout chromosome Omy19. Aquaculture.

[18] Wiens GD, Vallejo RL, Leeds TD, Palti Y, Hadidi S, Liu S (2013). Assessment of genetic correlation between bacterial cold water disease resistance and spleen index in a domesticated population of rainbow trout: identification of QTL on chromosome Omy19. PLoS One..

[19] Palti Y, Vallejo RL, Gao G, Liu S, Hernandez AG, Rexroad CE (2015). Detection and validation of QTL affecting bacterial cold water disease resistance in rainbow trout using restriction-site associated DNA sequencing. PLoS One.

[20] Vallejo RL, Liu S, Gao G, Fragomeni BO, Hernandez AG, Leeds TD (2017). Similar genetic architecture with shared and unique quantitative trait loci for bacterial cold water disease resistance in two rainbow trout breeding populations. Front Genet..

[21] Madsen L, Dalsgaard I (1999). Reproducible methods for experimental infection with *Flavobacterium psychrophilum* in rainbow trout *Oncorhynchus mykiss*. Dis Aquat Org..

[22] Garcia C, Pozet F, Michel C (2000). Standardization of experimental infection with *Flavobacterium psychrophilum*, the agent of rainbow trout *Oncorhynchus mykiss* fry syndrome. Dis Aquat Org..

[23] Monte M, Urquhart K, Secombes CJ, Collet B (2016). Individual monitoring of immune responses in rainbow trout after cohabitation and intraperitoneal injection challenge with *Yersinia ruckeri*. Fish Shellfish Immunol.

[24] Wargo AR, Kell AM, Scott RJ, Thorgaard GH, Kurath G (2012). Analysis of host genetic diversity and viral entry as sources of between-host variation in viral load. Virus Res.

[25] Kim SJ, Kim JO, Kim WS, Oh MJ (2016). Viral hemorrhagic septicemia virus (VHSV) infectivity dynamics in olive flounder, *Paralichthys olivaceus* with injection and immersion challenge routes. Aquaculture.

[26] Madejota J, Nyman P, Wiklun T (2000). *Flavobacterium psychrophilum*, invasion into and shedding by rainbow trout *Oncorhynchus mykiss*. Dis Aquat Org..

[27] Liu H, Izumi S, Wakabayashi H (2001). Detection of *Flavobacterium psychrophilum* in various organs of Ayu *Plecoglossus altivelis* by in situ hybridization. Fish Pathol..

[28] Amita K, Hoshino M, Honma T, Wakabayashi H (2000). An investigation on the distribution of *Flavobacterium psychrophilum* in the Umikawa River. Fish Pathol..

[29] Nematollahi A, Decostere A, Pasmans F, Ducatelle R, Haesebrouck F (2003). Adhesion of high and low virulence *Flavobacterium psychrophilum* strains to isolated gill arches of rainbow trout *Oncorhynchus mykiss*. Dis Aquat Org..

[30] Munang’andu HM, Evensen Ø (2015). A review of intra and extracellular antigen delivery systems for virus vaccines of finfish. J Immunol Res..

[31] Xu Z, Parra D, Gómez D, Salinas I, Zhang YA, von Gersdorff Jørgensens L (2013). Teleost skin, an ancient mucosal surface that elicits gut-like immune responses. Proc Natl Acad Sci USA.

[32] Salinas I (2015). The mucosal immune system of teleost fish. Biology..

[33] Jia R, Liu BL, Feng WR, Han C, Huang B, Lei JL (2016). Stress and immune responses in skin of turbot (*Scophthalmus maximum*) under different stocking densities. Fish Shellfish Immunol.

[34] Cordero H, Brinchmann MF, Cuesta A, Meseguer J, Esteban MA (2015). Skin mucus proteome map of European sea bass (*Dicentrarchus labrax*). Proteomics.

[35] Benhamed S, Guardiola FA, Mars M, Esteban MA (2014). Pathogen bacteria adhesion to skin mucus of fishes. Vet Microbiol.

[36] Lazado CC, Caipang CM (2014). Mucosal immunity and probiotics in fish. Fish Shellfish Immunol.

[37] Boutin S, Sauvage C, Bernatchez L, Audet C, Derome N (2014). Inter individual variation of the fish skin microbiota: host genetics basis of mutualism?. PLoS One.

[38] Guardiola FA, Cuesta A, Abellán E, Meseguer J, Esteban MA (2014). Comparative analysis of the humoral immunity of skin mucus from several marine teleost fish. Fish Shellfish Immunol.

[39] Pérez-Pascual D, Rochat T, Kerouault B, Gómez E, Neulat-Ripoll F, Henry C (2017). More than gliding: involvement of GldD and GldG in the virulence of *Flavobacterium psychrophilum*. Front Microbiol..

[40] Komen H, Thorgaard GH (2007). Androgenesis, gynogenesis and the production of clones in fishes: a review. Aquaculture.

[41] Seymour DK, Filiault DL, Henry IM, Monson-Miller J, Ravi M, Pang A (2012). Rapid creation of *Arabidopsis* doubled haploid lines for quantitative trait locus mapping. Proc Natl Acad Sci USA.

[42] Carlborg Ö, Haley CS (2004). Epistasis: too often neglected in complex traits studies?. Nat Rev Genet.

[43] Quillet E, Dorson M, Le Guillou S, Benmansour A, Boudinot P (2007). Wide range of susceptibility to rhabdoviruses in homozygous clones of rainbow trout. Fish Shellfish Immunol.

[44] Biacchesi S, Le Berre M, Le Guillou S, Benmansour A, Bremont M, Quillet E (2007). Fish genotype significantly influences susceptibility of juvenile rainbow trout, *Oncorhynchus mykiss* (Walbaum), to waterborne infection with infectious salmon anaemia virus. J Fish Dis.

[45] Verrier ER, Genet C, Laloë D, Jaffrezic F, Rau A, Esquerre D (2018). Genetic and transcriptomic analyses provide new insights on the early antiviral response to VHSV in resistant and susceptible rainbow trout. BMC Genomics.

[46] Langevin C, Blanco M, Martin SAM, Jouneau L, Bernardet JF, Houel A (2012). Transcriptional responses of resistant and susceptible fish clones to the bacterial pathogen *Flavobacterium psychrophilum*. PLoS One.

[47] Diter A, Quillet E, Chourrout D (1993). Suppression of first egg mitosis induced by heat shocks in the rainbow trout. J Fish Biol.

[48] Lynch M, Walsh B (1997). Genetics and analysis of quantitative trait.

[49] Palti Y, Gao G, Miller MR, Vallejo RL, Wheeler PA, Quillet E (2014). A resource of single-nucleotide polymorphisms for rainbow trout generated by restriction-site associated DNA sequencing of doubled haploids. Mol Ecol Resour..

[50] Duchaud E, Rochat T, Habib C, Barbier P, Loux V, Guérin C (2018). Genomic diversity and evolution of the fish pathogen *Flavobacterium psychrophilum*. Front Microbiol..

[51] Genotoul. http://get.genotoul.fr. Accessed 28 Jan. 2016.

[52] Baird NA, Etter PD, Atwood TS (2008). Rapid SNP discovery and genetic mapping using sequences RAD marker. PLoS One.

[53] Stacks. http://catchenlab.life.illinois.edu/stacks/. Accessed 09 June 2016.

[54] Catchen J, Hohenlohe P, Bassham S, Amores A, Cresko W (2013). Stacks: an analysis tool set for population genomics. Mol Ecol.

[55] Oral M. Insights into isogenic clonal fish line development using high-throughput sequencing technologies. University of Stirling. PhD thesis. 2016; http://hdl.handle.net/1893/24909.

[56] Sakamoto T, Danzmann RG, Gharbi K, Howard P, Ozaki A, Khoo SK (2000). A microsatellite linkage map of rainbow trout (*Oncorhynchus mykiss*) characterized by large sex-specific differences in recombination rates. Genetics.

[57] Anderson JL, Rodriguez Mari A, Braasch I, Amores A, Hohenlohe P, Batzel P (2012). Multiple sex-associated regions and a putative sex chromosome in zebrafish revealed by RAD mapping and population genomics. PLoS One.

[58] CarthaGène. http://www7.inra.fr/mia/T/CarthaGene/. Accessed on 02 Dec. 2016.

[59] de Givry S, Bouchez M, Chabrier P, Milan D, Schiex T (2005). CARTHA GENE: multipopulation integrated genetic and radiation hybrid mapping. Bioinformatics.

[60] Omyk_1.0. https://www.ncbi.nlm.nih.gov/assembly/GCF_002163495.1/. Accessed on 15 Sept 2017.

[61] Filangi O, Moreno C, Gilbert H, Legarra A, Le Roy P, Elsen JM. QTLMap, a software for QTL detection in outbred populations. In: Proceedings of the 9th world congress on genetics applied to livestock production, 1–6 Aug 2010, Leipzig; 2010.

[62] Elsen JM, Mangin B, Goffinet B, Boichard D, Le Roy P (1999). Alternative models for QTL detection in livestock. I. General Introduction. Genet Sel Evol..

[63] Le Roy P, Elsen JM, Boichard D, Mangin B, Bidanel JP, Goffinet B. An algorithm for QTL detection in mixture of full and half-sib families. In: Proceedings of the 6th world congress on genetics applied to livestock productions, 11–16 Jan 1998, Armidale; 1998.

[64] R Development Core Team. R: a language and environment for statistical computing. R Foundation for Statistical Computing, Vienna, Austria. ISBN. 2013. https://www.r-project.org/. Accessed 11 Jan. 2017.

[65] Moreno CR, Elsen JM, Le Roy P, Ducrocq V (2005). Interval mapping methods for detecting QTL affecting survival and time-to-event phenotypes. Genet Res.

[66] Cox DR (1972). Regression models and life-tables (with discussion). J R Stat Soc Ser B Stat Methodol..

[67] Benjamini Y, Hochberg Y (1995). Controlling the false discovery rate: a practical and powerful approach to multiple testing. J R Stat Soc Ser B Stat Methodol..

[68] Kause A, Ødegard J (2012). The genetic analysis of tolerance to infections: a review. Front Genet..

[69] Harrell FE, Davis CE (1982). A new distribution-free quantile estimator. Biometrika.

[70] Li H (2011). A quick method to calculate QTL confidence interval. J Genet..

[71] Phillips RB, Nichols KM, DeKoning JJ, Morasch MR, Keatley KA, Rexroad C (2006). Assignment of rainbow trout linkage groups to specific chromosomes. Genetics.

[72] Guyomard R, Boussaha M, Krieg F, Hervet C, Quillet E (2012). A synthetic rainbow trout linkage map provides new insights into the salmonid whole genome duplication and the conservation of synteny among teleosts. BMC Genet.

[73] Suppl Langevin C (2012). Figure 1. This excel file contains the complete list of up- and down- regulated genes in rainbow trout clonal lines B57_s and A3_r following *F. psychrophilum* JIP 02/86 infection. PLoS One.

[74] Langevin C (2012). Suppl. Figure 2. This table contains the list of probes for wich up- or down- regulation was significant in only one of the two fish clonal lines, while a high adj. *p* value in the other line indicated a large variation of the expression level. PLoS One.

[75] NCBI Oncorhynchus mykiss Annotation Release 100. https://www.ncbi.nlm.nih.gov/genome/annotation_euk/Oncorhynchus_mykiss/100/. Accessed 12 Dec 2017.

[76] Castro R, Jouneau L, Tacchi L, Macqueen DJ, Alzaid A, Secombes CJ, Martin SAM, Boudinot P (2015). Disparate developmental patterns of immune responses to bacterial and viral infections in fish. Sci Rep..

[77] Flint J, Mott R (2001). Finding the molecular basis of quantitative traits: successes and pitfalls. Nat Rev Genet.

[78] Carlborg Ö, Brockmann GA, Haley CS (2005). Simultaneous mapping of epistatic QTL in Du6i x DBA/2 mice. Mamm Genome.

[79] Carlborg Ö, Kerje S, Schütz K, Jacobsson L, Jensen P, Andersson L (2003). A global search reveals epistatic interaction between QTL for early growth in the chicken. Genome Res.

[80] Fijneman RJ, de Vries SS, Jansen RC, Demant P (1996). Complex interactions of new quantitative trait loci, Sluc1, Sluc2, Sluc3, and Sluc4, that influence the susceptibility to lung cancer in the mouse. Nat Genet.

[81] van Wezel T, Stassen AP, Moen CJ, Hart AA, van der Valk MA, Demant P (1996). Gene interaction and single gene effects in colon tumour susceptibility in mice. Nat Genet.

[82] Rochat T, Fujiwara-Nagata E, Calvez S, Dalsgaard I, Madsen L, Calteau A (2017). Genomic characterization of *Flavobacterium psychrophilum* serotypes and development of a multiplex PCR-Based serotyping scheme. Front Microbiol..

