# Correction to: Quantitative trait loci for resistance to *Flavobacterium psychrophilum* in rainbow trout: effect of the mode of infection and evidence of epistatic interactions

**DOI:** 10.1186/s12711-019-0451-0

**Published:** 2019-03-20

**Authors:** Clémence Fraslin, Nicolas Dechamp, Maria Bernard, Francine Krieg, Caroline Hervet, René Guyomard, Diane Esquerré, Johanna Barbieri, Claire Kuchly, Eric Duchaud, Pierre Boudinot, Tatiana Rochat, Jean-François Bernardet, Edwige Quillet

**Affiliations:** 1 GABI, INRA, AgroParisTech, Université Paris-Saclay, 78350 Jouy-en-Josas, France; 2 SYSAAF Section Aquacole, Campus de Beaulieu, 35000 Rennes, France; 3 GABI, SIGENAE, INRA, AgroParisTech, Université Paris-Saclay, 78350 Jouy-en-Josas, France; 4 GeT-PlaGe, Genotoul, INRA US1426, 31320 Castanet-Tolosan Cedex, France; 5 Virologie et Immunologie Moléculaires, INRA, Université Paris-Saclay, 78350 Jouy-en-Josas, France; 6 Present Address: BIOEPAR, INRA, Oniris, Université Bretagne Loire, 44307 Nantes, France

## Correction to: Fraslin et al. Genet Sel Evol (2018) 50:60 10.1186/s12711-018-0431-9

After publication of this work [1], we noted that there was an error in Table 3 Line 4: ^b^Omy2_**Omy3**_ should be ^b^Omy21_**Omy3**_.

The correct Table 3 is included here.

**Table 3:** Results of QTL analysis using the model *M2* for resistance trait following injection or immersion challenges

Infection route	QTL	LRTmax	Position (cM)	CI (95%)	Increase in survival rate		Resistance origin		*P* value fixed effect	*P* value interaction
					Fixed_R (%)	Fixed_S (%)	Fixed_R	Fixed_S		
IMMERSION	Omy17_**Omy3**_	13.97*	61	0–92	38	7	AP2	AP2	***	NS
	Omy25a_**Omy3**_	10.41*	4	0–35	10	18	B57	B57	***	NS
*Type 1 interaction*										
INJECTION	^a^Omy3_**Omy29**_	15.27**	89	46–105	16	47	AP2	AP2	***	***
IMMERSION	^b^Omy21_**Omy3**_	15.35**	97	63–104	4	39	B57	B57	***	***
	^b^Omy3_**Omy21**_	40.73***	87	82–93	20	55	AP2	AP2	***	***
	^c^Omy3_**Omy2**_	35.66***	87	81–94	17	44	AP2	AP2	***	***
INJECTION	^a^Omy29.2_**Omy3**_	14.85*	23	8–49	5	48	B57	AP2	***	*
	Omy17_**Omy25a**_	15.85**	73	53–79	11	53	AP2	B57	***	***
IMMERSION	Omy7.2_**Omy21**_	11.48*	7	0–103	5	31	AP2	B57	***	***
*Type 2 interaction*										
INJECTION	^d^Omy25a_**Omy3**_	25.49***	14	10–18	53	16	B57	B57	***	*
	^d^Omy3_**Omy25a**_	35.35***	89	86–92	59	22	AP2	AP2	***	***
	Omy26_**Omy29**_	11.75*	18	0–34	30	26	AP2	AP2	***	***
INJECTION	Omy17_**Omy29**_	18.29***	74	58–92	47	11	AP2	B57	***	***
IMMERSION	Omy24_**Omy2**_	12.71*	4	0–19	20	1	B57	AP2	***	***
*Type 3 interaction*										
IMMERSION	Omy7.1_**Omy2**_	16.42**	61	32–87	19	19	B57	AP2	***	***

The table presents chromosome-wide or genome-wide significant QTL detected for STATUS using model *M*2; Reciprocal interactions could be tested only for QTL detected in the first STATUS analysis (model *M*1); LRTmax = maximum of likelihood ratio test; Position in the genetic map in centimorgans (cM); CI = confidence interval; Chromosome-wide significant = *P ≤ 0.01; Genome-wide significant = **P ≤ 0.05 or ***P ≤ 0.01; *P* values for fixed effect and interaction corrected with Benjamini–Hochberg method: Non-significant = NS; *P value ≤ 0.05; ***P value ≤ 0.001

^a^The reciprocal interaction could not be tested as a new QTL (Omy29.2_**Omy3**_-QTL) was detected with the reciprocal model

^b,d^Reciprocal models for QTL pairs

^c^The QTL in the reciprocal model (Omy2_**Omy3**_-QTL) was only suggestive (*P* ≤ 0.05) at the chromosome-wide level

## References

[1] Fraslin C (2018). Quantitative trait loci for resistance to *Flavobacterium psychrophilum* in rainbow trout: effect of the mode of infection and evidence of epistatic interactions. Genet Sel Evol.

