# Supplementary figures and images for: Quantitative trait loci for resistance to Flavobacterium psychrophilum in rainbow trout: effect of the mode of infection and evidence of epistatic interactions
Source: Genet Sel Evol. 2018 Nov 16;50:60. doi: 10.1186/s12711-018-0431-9 (PMC6240304; doi:10.1186/s12711-018-0431-9)

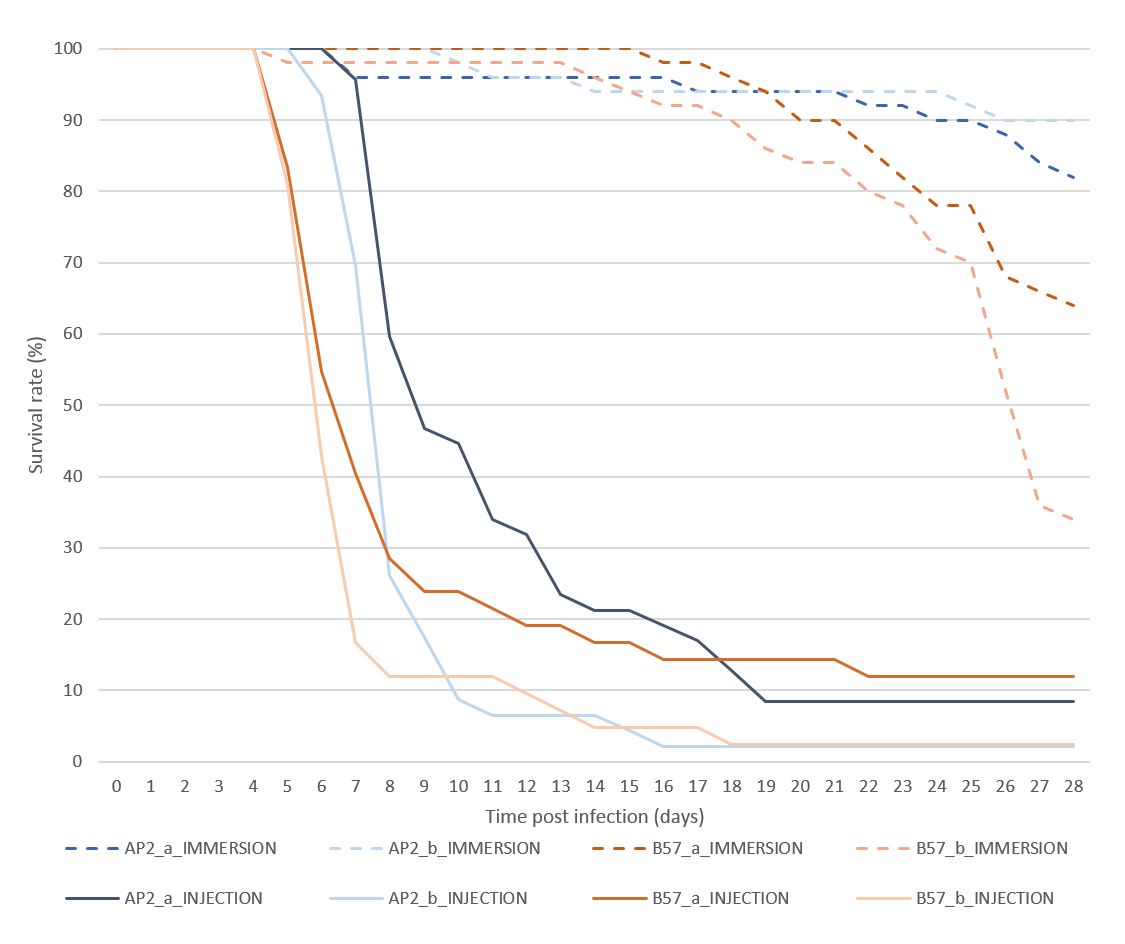

Supplement: Supplementary file 1 — Additional file 1: Figure S1. Cumulative survival curves of fish from AP2 and B57 grandparental isogenic lines infected with Flavobacterium psychrophilum. Description: Fish were infected with the F. psychrophilum FRGDSA 1882/11 strain and mortality was recorded for 29 days post-infection. For injection protocol (—), 100 fish (average weight of 10.3 g for AP2 and 12.2 g for B57) were infected with (a): 450 CFU/mL (replicate of 50 fish) or (b): 300–550 CFU/mL (replicate of 50 fish). For the immersion protocol (- - - - -), 100 fish (average weight of 1.1 g for AP2 fish and 1.0 g for B57 fish) were infected by immersion for 4 h in a bacterial suspension (approximately 3.107 CFU/mL) in static water maintained at 10 °C with vigorous aeration, in two replicates of 50 fish each (a) and (b). [file 12711_2018_431_MOESM1_ESM.png]

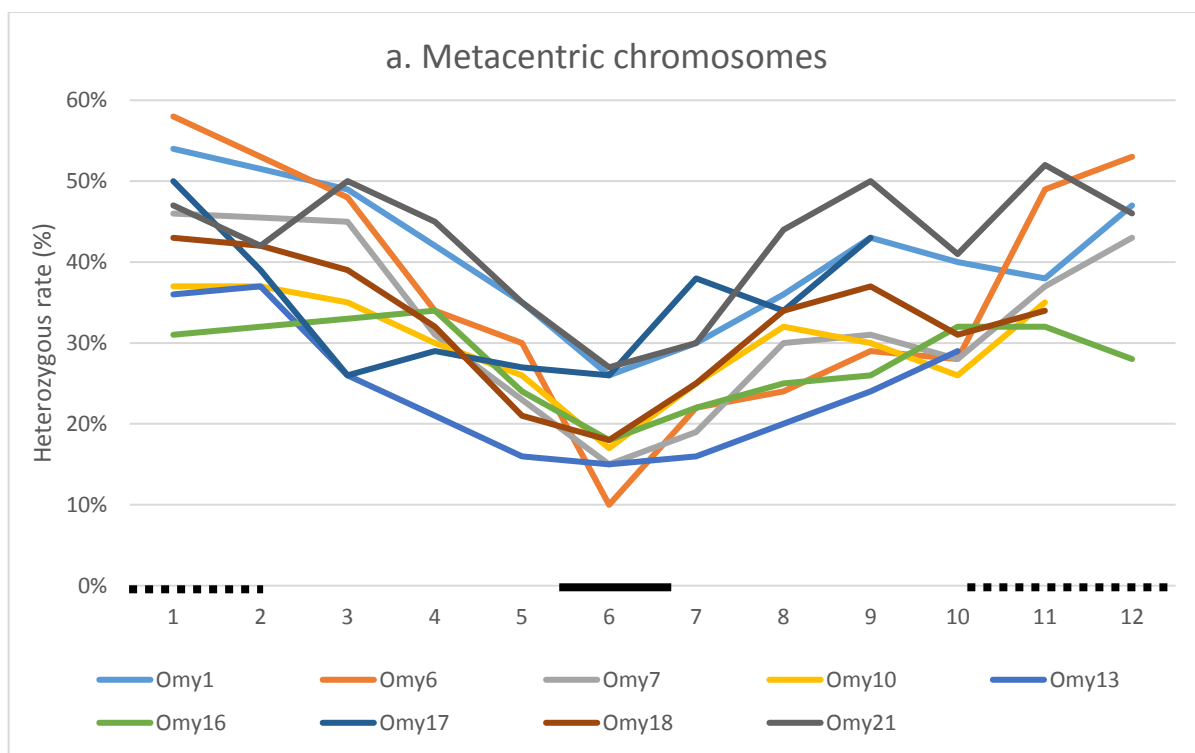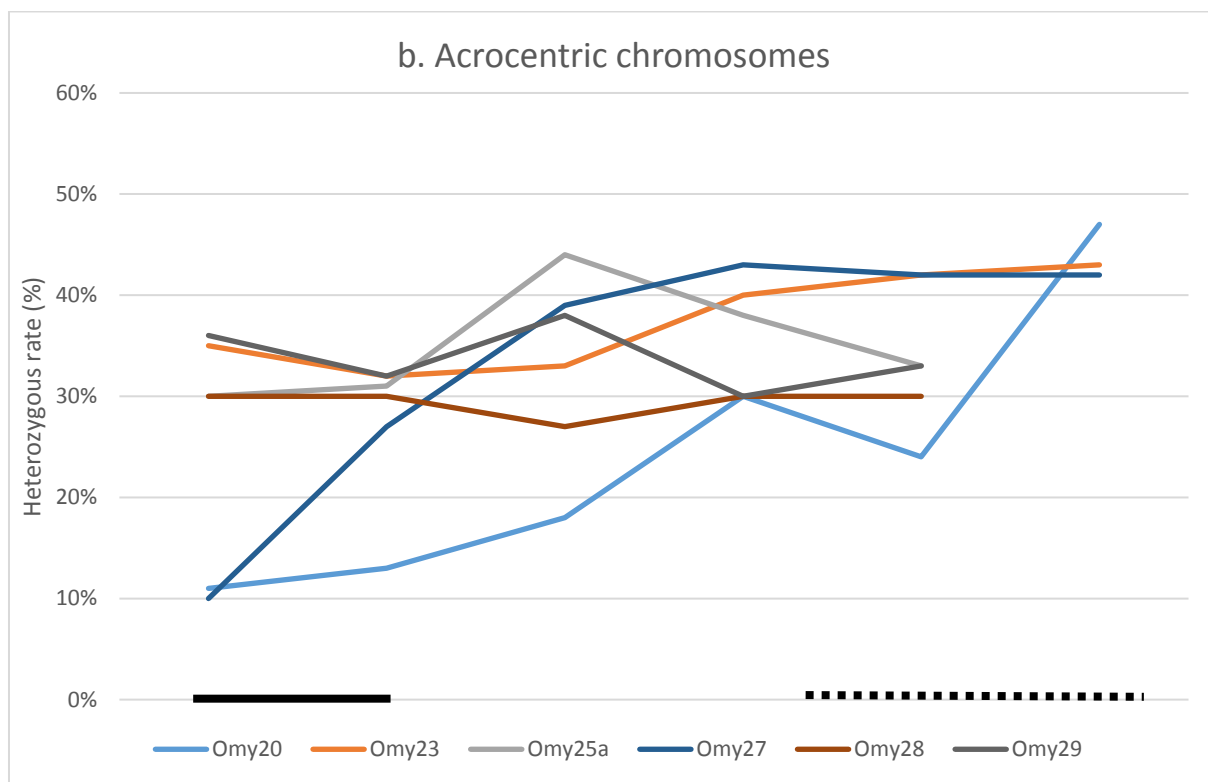

Supplement: Supplementary file 3 — Additional file 3: Figure S2. Evolution of the rate of hererozygosity along chromosomes (mean values for the 24 individuals with an overall rate of heterozygosity higher than 1%). Description: Metacentric chromosomes (a): under the hypothesis of spontaneous retention of the second polar body during meiosis, the rate of heterozygosity is expected to be lower around the centromere (—) than in telomeric regions (- - - -). Acrocentric chromosomes (b): under the hypothesis of spontaneous retention of the second polar body during meiosis, the rate of heterozygosity is expected to increase along the chromosome from the centromeric region to the telomere. Data are illustrated for 15 chromosomes. [file 12711_2018_431_MOESM3_ESM.pdf]

Type 1 interaction

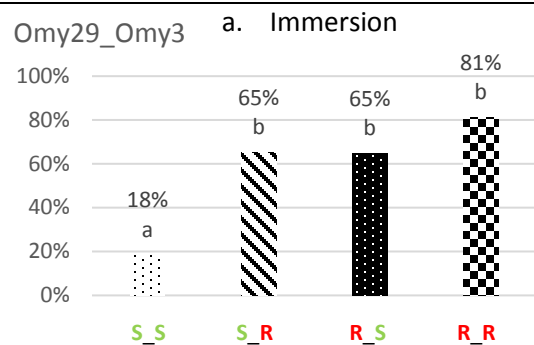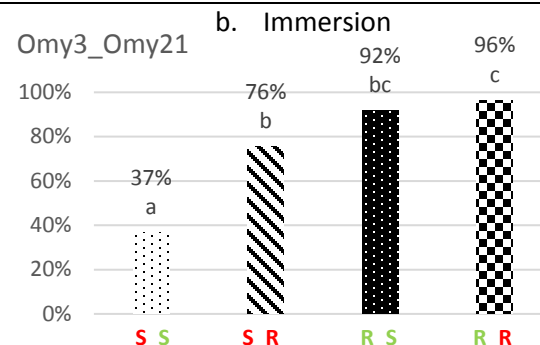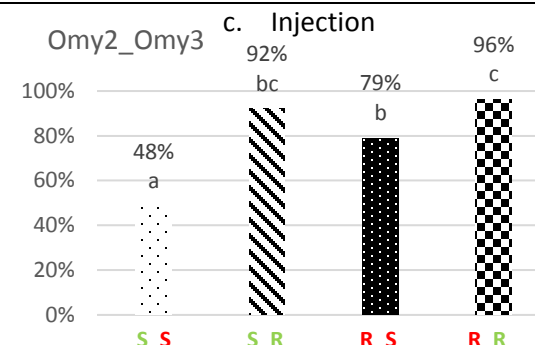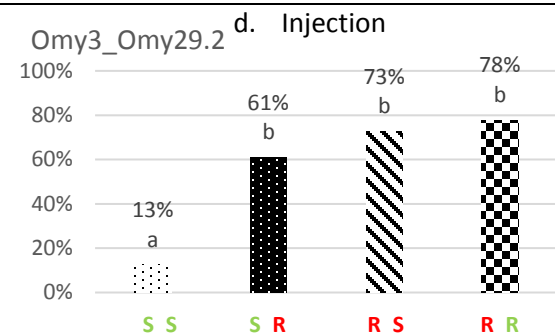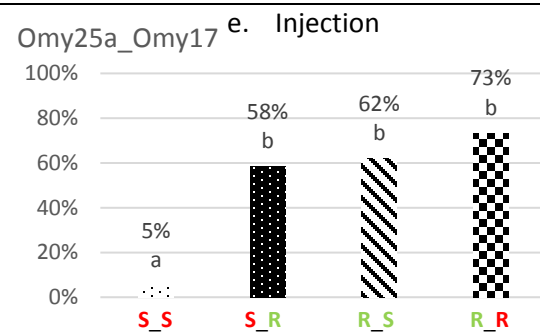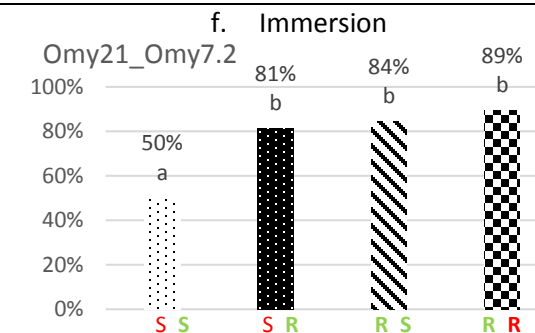

Type 2 interaction

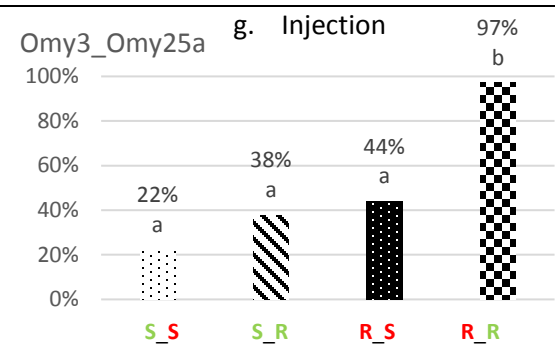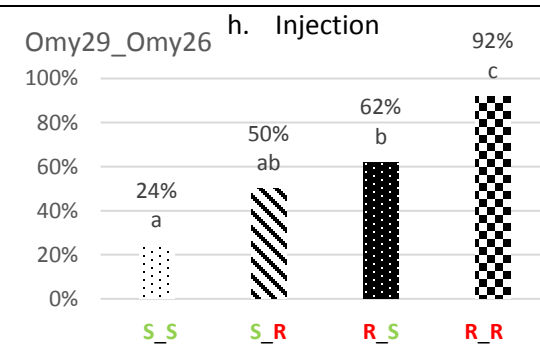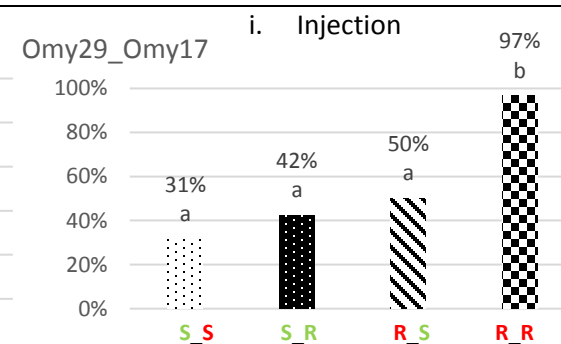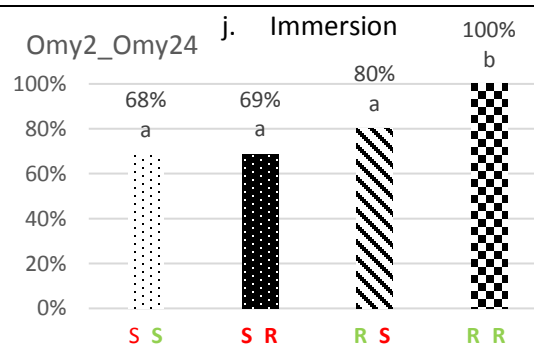

Type 3 interaction

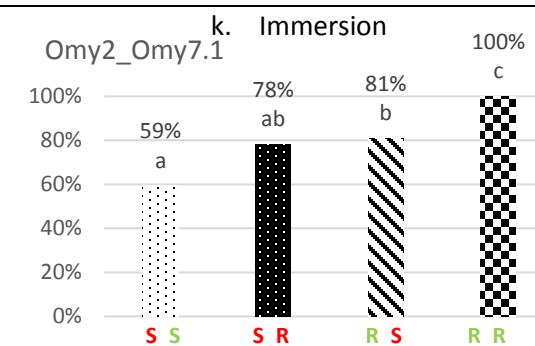

Supplement: Supplementary file 5 — Additional file 5: Figure S4. Final survival rate according to the allele origin at pairs of epistatic QTL for resistance to infection with F. psychrophilum. Description: For each figure, abscissa corresponds to the combination of favourable (R) and unfavourable (S) alleles with the grandparent origin in colour (green for B57 and red for AP2) for each pair of epistatic QTL OmyA_OmyB. Survival rates (in ordinate) with similar letters are not significantly different (Fisher exact test P ≤ 0.05 and Benjamini–Hochberg correction for multiple testing of stat package from R software). [file 12711_2018_431_MOESM5_ESM.pdf]
