# Supplementary material for: Quantitative trait loci for resistance to Flavobacterium psychrophilum in rainbow trout: effect of the mode of infection and evidence of epistatic interactions
Source: Genet Sel Evol. 2018 Nov 16;50:60. doi: 10.1186/s12711-018-0431-9 (PMC6240304; doi:10.1186/s12711-018-0431-9)
Supplement: Supplementary file 4 — Additional file 4: Figure S3. Graphical compilation of likelihood ratio profiles calculated for each chromosome (1-cM interval) for the two resistance traits after the two modes of infection challenges. Description: (a) RESISTANCE and (b) STATUS after injection challenge, (c) RESISTANCE and (d) STATUS after immersion challenge. For each chromosome, horizontal bars indicate the corresponding significance thresholds (green: P ≤ 0.01 at the chromosome-wide level; red: P ≤ 0.05 at the genome-wide level). [file 12711_2018_431_MOESM4_ESM.pdf]

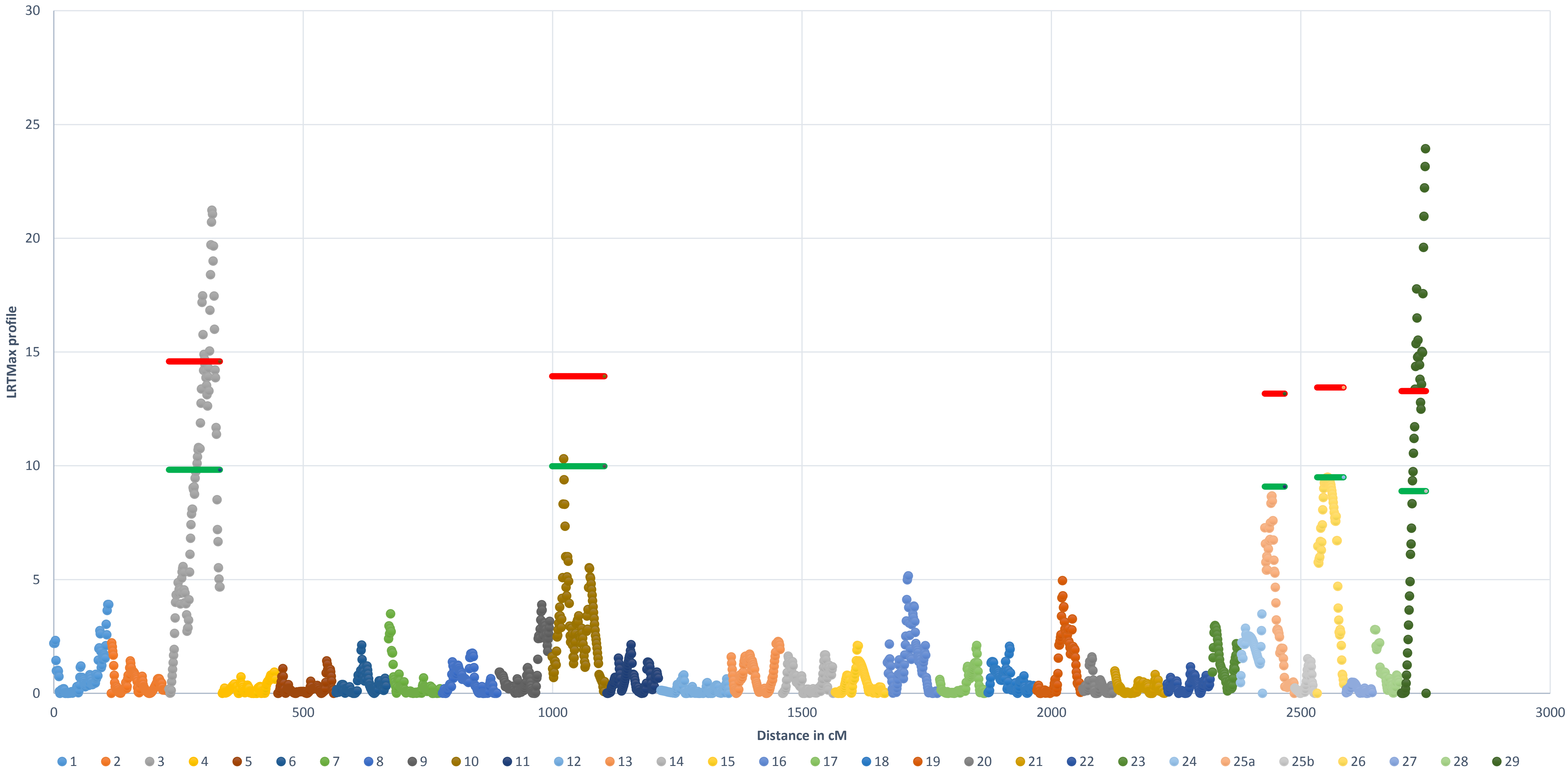

Additional file 4: Figure S3.a Graphical compilation of likelihood ratio profiles calculated for each chromosome (1cM interval) for RESISTANCE after the injection challenge

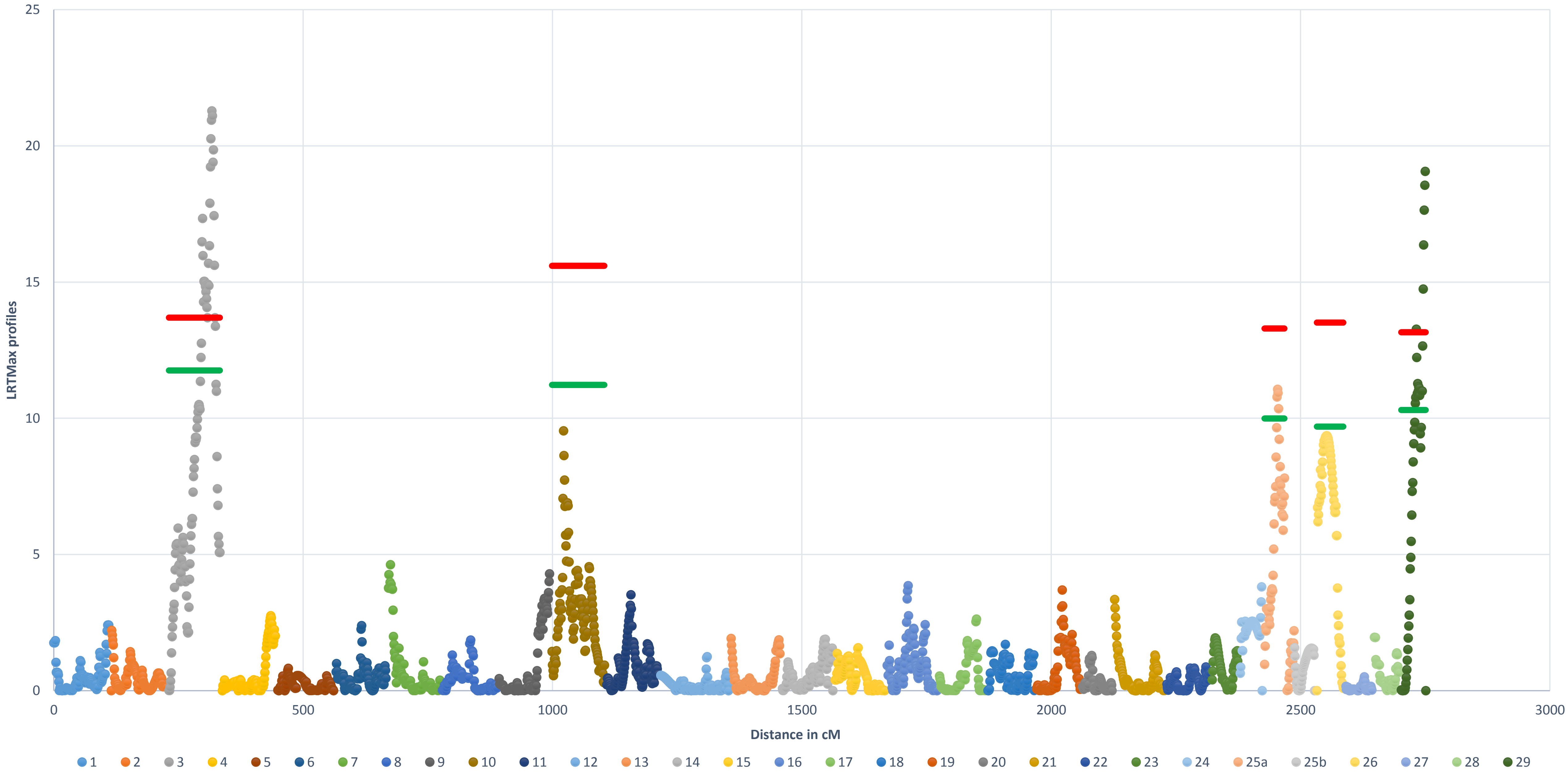

Additional file 4: Figure S3.b Graphical compilation of likelihood ratio profiles calculated for each chromosome (1cM interval) for STATUS after the injection challenge

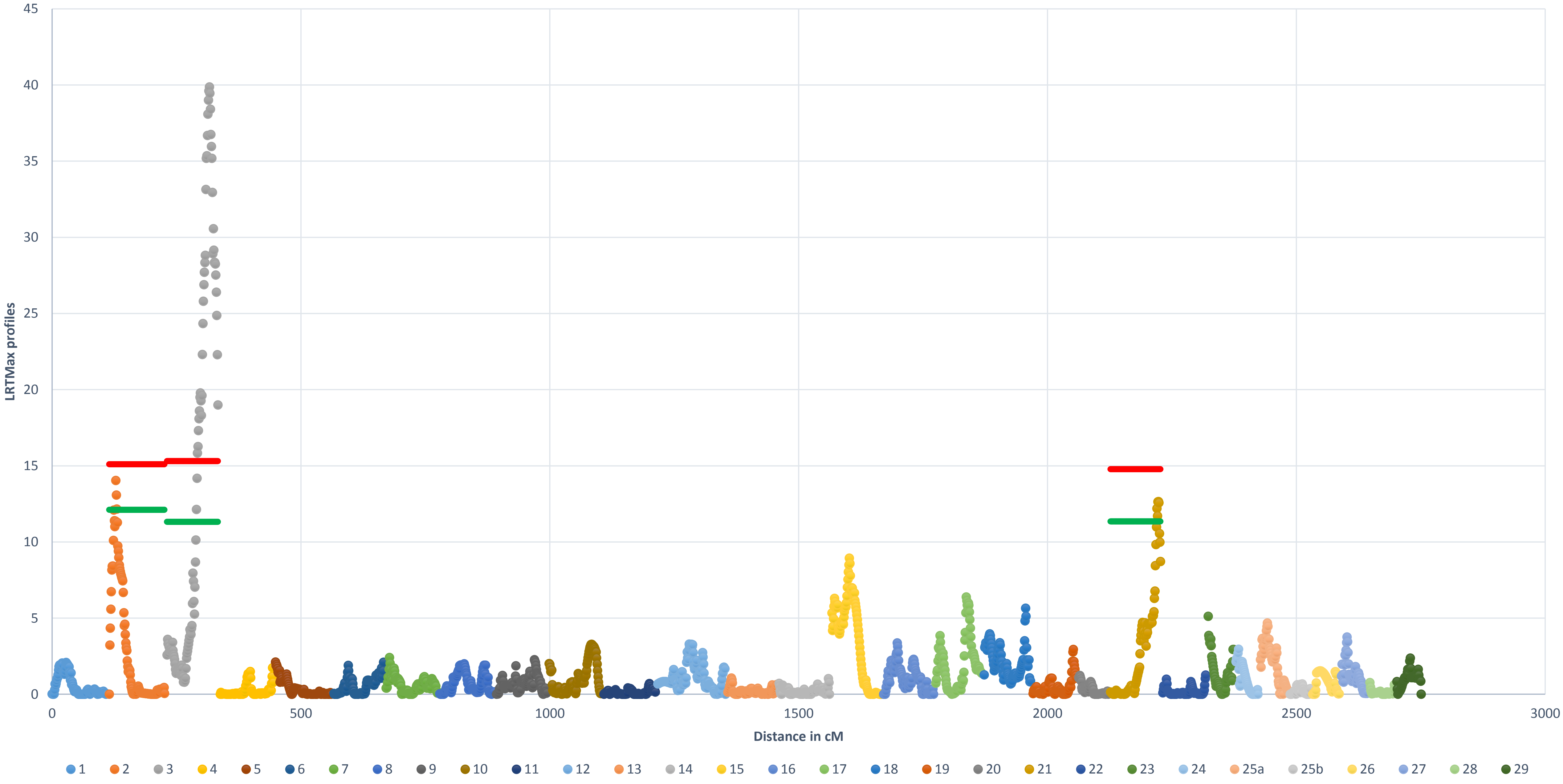

Additional file 4: Figure S3.c Graphical compilation of likelihood ratio profiles calculated for each chromosome (1cM interval) for RESISTANCE after the immersion challenge

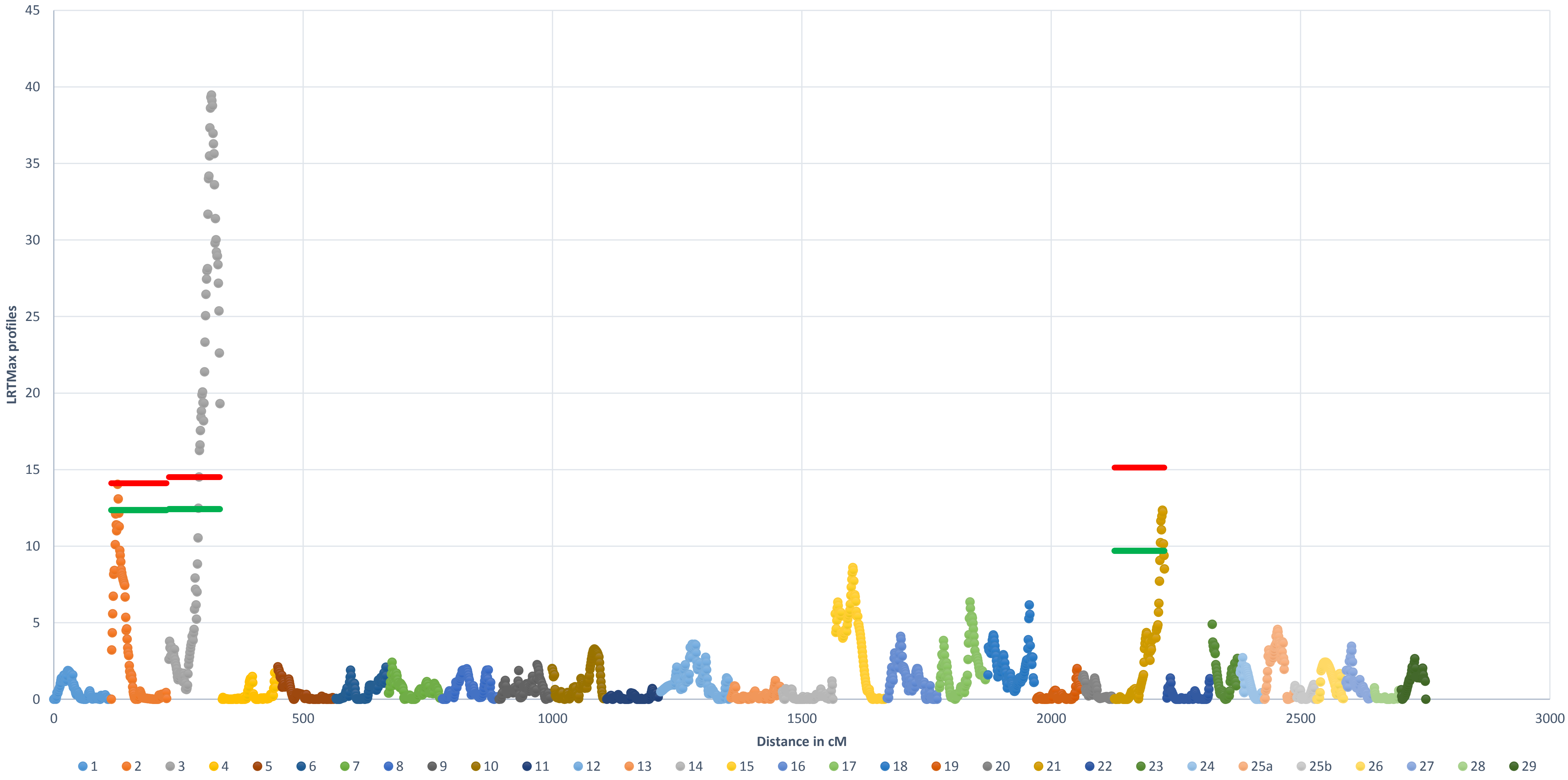

Additional file 4: Figure S3.d Graphical compilation of likelihood ratio profiles calculated for each chromosome (1cM interval) for STATUS after the immersion challenge
