# Supplementary material for: Quantitative trait loci for resistance to Flavobacterium psychrophilum in rainbow trout: effect of the mode of infection and evidence of epistatic interactions
Source: Genet Sel Evol. 2018 Nov 16;50:60. doi: 10.1186/s12711-018-0431-9 (PMC6240304; doi:10.1186/s12711-018-0431-9)
Supplement: Supplementary file 6 — Additional file 6. Details on the immune genes induced by F. psychrophilum infection that map within resistance-associated QTL. [file 12711_2018_431_MOESM6_ESM.pdf]

Details on the immune genes induced by *F. psychrophilum* infection that map within resistance-associated QTL

In a previous study [1] we analysed the transcriptome response to Fp in the pronephros of two trout isogenic lines (B57 and A3) with contrasted susceptibility to Fp, using micro-arrays. A list of 571 genes differentially expressed after Fp injection in at least one of these lines was generated ([1] supplementary material [2, 3]). All probes corresponding to these genes were mapped on the rainbow trout genome [4], to test whether differentially expressed genes would be located close to a QTL, and to build a preliminary list of potentially relevant Fp induced genes combining expression and positional features of interest. Probes positions were compared to the rainbow trout annotation [5] to name the corresponding protein. Sixty four probes (corresponding to 49 genes) were located within or close to the 95% confidence interval of QTL detected in the present study (see Table a, Additional file 2: Tables S4 and S5).

Among those forty-nine genes, fourteen had functions suggesting that they might be directly implicated in the resistance controlled by the different QTL in (close to) which they are located (Table a). These genes can be classified in four functional categories: (1) bacterial sensors and damage associated molecular pattern (*DAMP*) molecules ; (2) inflammatory factors; (3) effectors killing bacteria or affecting the host; and (4) Interferon stimulated genes (ISG).

Table a. List of functional genes positioned inside or very close to the 95% confidence interval of the QTL associated with resistance to Fp

Chromosomes	Protein name	Functional categories	Function
Omy2	Steap4/ Tumor Necrosis Factor, Alpha-Induced Protein 9	2	A metalloreductase. Plays a role in the regulation of inflammatory cytokines and NF-κB signalling.
Omy2	complement C3	3	C3 plays a central role in the activation of the complement system known as essential to the innate immunity.
Omy2	Hepcidin	3	Antimicrobial peptide induced after bacterial infection playing an important role in regulating the systematic iron homeostasis.
Omy2	C-type lectin domain family 4 member E-like/Macrophage-inducible C-type lectin/CLEC4-like	1	Cell-surface receptor for mycobacteria and other pathogens, induces inflammatory cytokines via the NF-κB pathway.
Omy2	C-type lectin B/Clec4-like	1	Endocytic receptor, pro-inflammatory.
Omy3	CD209/Clec4-like	1	Pathogen-recognition receptor expressed on the surface of immature dendritic cells and involved in initiation of primary immune response.
Omy3	interleukin-1 receptor type 2 (Il1r2)	2	Receptor of interleukin-1, a major pro-inflammatory cytokine.
Omy7	Differentially regulated trout protein (Drtp1)	2	Acute phase protein induced by polyinosinic:polycytidylic acid (polyIC), lipopolysaccharide (LPS) and tumor necrosis factor-alpha (TNFα)
Omy7	Mmp13/collagenase 3	3	A metalloprotease. Plays a key role in degradation and remodelling of host extracellular matrix proteins.
Omy10	VHSV induced gene -2	4	Type I IFN inducible transcript.
Omy17	Ifi44	4	Interferon stimulated gene.
Omy17	Ifitm-like	4	Interferon stimulated gene with antiviral activity, prevents viral fusion and release of viral contents into the cytosol.
Omy17	Mmp13/collagenase 3	3	A metalloprotease. Plays a key role in degradation and remodelling of host extracellular matrix proteins.
Omy21	Toll-like receptor 2 (Tlr2)	1	A key bacterial sensor. Recognizes LPS, lipoteichoic acid, and lipoproteins, also β-glucans and a few viral glycoproteins.
Omy29	High mobility group Box3 (Hmgb3)	1	Associated with nuclear chromatin or present in the cytosol. May also function as danger signal and trigger host immune activation when released from dead cells.

(1) The initiation of the antibacterial response is an obvious level at which genetic variation might condition the efficiency of the defence reaction of the host. CD209/DC-SIGN and other related lectins recognises mannose carbohydrates, which are important molecular patterns expressed by pathogens, especially bacteria. In mammals, this interaction activates phagocytosis of macrophage [6], while on dendritic cells CD209 activates CD4+ T cells. TLR2 is another key sensor of bacterial pathogens, binding for example LPS and lipoteichoic acids. The HMGB3-like may not be a true sensor, but Hmgb proteins are inflammatory proteins also present in plants; they are expected to bind various pathogen associated molecular patterns when released from dead cells, and send strong activation signals that can amplify the immune response.

(2) IL1 is one of the most important pro-inflammatory cytokines, and its receptor is a key element of the downstream signalling; in contrast, the metalloreductase Steap4, as known as TNFα-induced protein 9, regulates degradation of the NF-κB inhibitory molecule IκBα and phospho-STAT3, appearing as a negative regulator of inflammation. Thus, these two genes are important factors of the main inflammatory axes based on the cytokines IL1 and TNFα. The function of the acute phase protein Drtp1 remains unknown, but it is encoded by one of the most induced gene after polyIC, LPS or TNFa treatment, underscoring its potential importance as an inflammatory factor.

(3) The two important antibacterial effectors located close to resistance QTL regions comprise a C3 protein of the complement cascade and the antibacterial peptide Hepcidin. Besides, the metalloprotease Mmp13, typically induced by pro-inflammatory cytokines, is implicated in the degradation of the extracellular matrix and in tissue remodelling [7].

(4) Finally, it is interesting to note that several important ISG (vig2, IFI44, IFITM) also were upregulated by the bacterial infection. They are all located in the same genomic region linked to Omy17. They modulate multiple pathways of inflammation, which can be either favourable of detrimental to the host depending on the strength and context of the response.

The genes *steap4*, *c3*, *hamp*, and *tlr2* were all well up-regulated in the susceptible line B57 compared to the resistant line in our transcriptome analysis [1]. Interestingly, they are located in two QTL (Omy2 and Omy21), for which the favourable allele origin was from the grandparent line B57, suggesting that higher induction of those genes might favour resistance in this case.

References

[1] Langevin C, Blanco M, Martin SAM, Jouneau L, Bernardet JF, Houel A, et al. Transcriptional responses of resistant and susceptible fish clones to the bacterial pathogen Flavobacterium psychrophilum. PLoS One. 2012;7:e39126.

[2] Langevin C. Suppl. Figure 1. This excel file contains the complete list of up- and down- regulated genes in rainbow trout clonal lines B57_s and A3_r following F. psychrophilum JIP 02/86 infection. 2013. PLoS One. 2012;7:e39126.s001. Accessed 2 Nov. 2017

[3] Langevin C. Suppl. Figure 2. This table contains the list of probes for wich up- or down- regulation was significant in only one of the two fish clonal lines, while a high adj. p value in the other line indicated a large variation of the expression level. PLoS One. 2012;7:e39126.s002. Accessed 2 Nov. 2017

[4] Omyk_1.0: https://www.ncbi.nlm.nih.gov/assembly/GCF_002163495.1/ Accessed on 15 Sept. 2017.

[5] NCBI Oncorhynchus mykiss Annotation Release 100 : https://www.ncbi.nlm.nih.gov/genome/annotation_euk/Oncorhynchus_mykiss/100/ . Accessed 12 Dec. 2017

[6] McGreal E, Miller J, Gordon S. Ligand recognition by antigen-presenting cell C-type lectin receptors. Curr. Opin. Immunol. 2005;10.1016/j.coi.2004.12.001

[7] Goldring MB, Otero M, Plumb DA, et al. Roles of inflammatory and anabolic cytokines in cartilage metabolism: signals and multiple effectors converge upon mmp-13 regulation in osteoarthritis. Eur. Cell Mater. 2011; 10.22203/eCM.v021a16.
